# Poly (ADP-ribose) polymerase 1 promotes HuR/ELAVL1 cytoplasmic localization and inflammatory gene expression by regulating p38 MAPK activity

**DOI:** 10.1007/s00018-024-05292-2

**Published:** 2024-06-09

**Authors:** Xingyue Fu, Jiaqi Zhang, Keke Sun, Meiqi Zhang, Shuyan Wang, Meng Yuan, Wenguang Liu, Xianlu Zeng, Xueqing Ba, Yueshuang Ke

**Affiliations:** The Key Laboratory of Molecular Epigenetics of the Ministry of Education, School of Life Science, Northeast Normal University, Changchun, 130024 Jilin China

**Keywords:** HuR, Inflammation, PARP1, p38, mRNA stability

## Abstract

Post-transcriptional regulation of cytokine/chemokine mRNA turnover is critical for immune processes and contributes to the mammalian cellular response to diverse inflammatory stimuli. The ubiquitous RNA-binding protein human antigen R (HuR) is an integral regulator of inflammation-associated mRNA fate. HuR function is regulated by various post-translational modifications that alter its subcellular localization and ability to stabilize target mRNAs. Both poly (ADP-ribose) polymerase 1 (PARP1) and p38 mitogen-activated protein kinases (MAPKs) have been reported to regulate the biological function of HuR, but their specific regulatory and crosstalk mechanisms remain unclear. In this study, we show that PARP1 acts via p38 to synergistically promote cytoplasmic accumulation of HuR and stabilization of inflammation-associated mRNAs in cells under inflammatory conditions. Specifically, p38 binds to auto-poly ADP-ribosylated (PARylated) PARP1 resulting in the covalent PARylation of p38 by PARP1, thereby promoting the retention and activity of p38 in the nucleus. In addition, PARylation of HuR facilitates the phosphorylation of HuR at the serine 197 site mediated by p38, which then increases the translocation of HuR to the cytoplasm, ultimately stabilizing the inflammation-associated mRNA expression at the post-transcriptional level.

**Supplementary Information:**

The online version contains supplementary material available at 10.1007/s00018-024-05292-2.

## Introduction

The RNA binding protein human antigen R (HuR)/embryonic lethal abnormal vision-like (ELAVL1) is a member of the Hu/ELAV family and is widely expressed in eukaryotic cells [1]. Although HuR is predominantly localized in the nucleus, HuR is known to shuttle from the nucleus to the cytoplasm, where it protects the stability of target mRNAs and facilitates translation [2]. HuR mainly binds to transcripts at AU-rich elements (AREs) that are often located in the 3'-untranslated region of target mRNAs. HuR targets include mRNAs that encode proteins associated with cell proliferation, aging and stress [1]. Especially, HuR is responsible for the stability of many inflammatory genes [1, 3, 4]. Thus, HuR plays a key role in the progression of many chronic inflammatory diseases and various types of cancers [1–7].

The function of HuR is inextricably linked to its localization, which is regulated by the binding of HuR to various kinases, which then mediate post-translational modifications (PTMs) [1]. Previously, we have shown that poly (ADP-ribose) polymerase 1 (PARP1) binds to HuR and catalyzes the poly ADP-ribosylation (PARylation) of HuR under inflammatory conditions [8–10]. PARylation is one of an essential PTM in cells that is catalyzed by the PARP family of enzymes that use NAD ^+^ as a substrate to synthesize poly ADP-ribose (PAR) units in linear or branched chains and bind them to acceptor proteins [11]. As the most abundant and ubiquitous member of the PARP family, PARP1 accounts for approximately 90% of the PARP activity [12, 13]. We have previously shown that PARP1 activation contributes to the PARylation of HuR, specifically at the aspartic acid 226 (D226) site, which further promotes HuR migration from the nucleus to the cytoplasm. The accumulation of HuR or HuR-mRNA complexes in the cytoplasm leads to an increase in inflammation-associated mRNA stability [9, 14]. However, the mechanisms through which PARP1 mediates the translocation of HuR remain unclear.

In addition to PARylation, phosphorylation of HuR mediated by various phosphokinases has received extensive attention due to its influence on the affinity of HuR to RNA and the distribution of HuR in cells [1, 15–18]. For example, phosphorylation of HuR by p38 mitogen-activated protein kinase (MAPK) leads to the accumulation of HuR in the cytoplasm and increased HuR binding to target mRNAs [15, 16]. Several studies have indicated that HuR may be a key effector of p38-mediated functions in the inflammatory response, but the influence and mechanism of p38 on HuR is not fully clear [15, 16, 19]. Interestingly, p38 activation is reportedly impaired in PARP1^−/−^ glial cells in response to inflammatory stimuli, while PARP1 has been shown to regulate the mRNA expression of inflammatory gene IP-10 via modulating the p38 signaling pathway [20, 21]. Together, these findings suggest that the interaction between PARP1 and p38 is important in the regulation of inflammatory gene expression, although the mechanism remains unclear. Here, we sought to determine the roles of PARP1 and p38 in inflammatory gene expression at the post-transcriptional level via regulation of the localization or function of HuR.

We found that PARP1 and p38 synergistically promote the accumulation of HuR in the cytoplasm and subsequently increase the stability of inflammation-associated mRNAs. Specifically, tumor necrosis factor-a (TNFα) stimulation promotes the recruitment of p38 to the PAR chain of PARP1. PARP1 then PARylates p38, which further increases the activation of p38. Meanwhile, PARylation of HuR at D226 facilitates p38 to phosphorylate HuR at the serine197 (S197) site, which leads to increased distribution of HuR in the cytoplasm and stabilization of target mRNAs. Our findings highlight a novel function of the PARP1-p38 axis as a regulator of HuR function, and further our understanding of the mechanism by which PARP1 and p38 synergistically regulate the stability of inflammatory factors at the post-transcriptional level in response to inflammatory stimuli.

## Materials and methods

### Antibodies

Mouse mAb against HuR (1: 3000, 3A2, sc-5261), PARP1 (1: 3000, B-10, sc-74470), and Histone H1 (1: 1000, AE-4, sc-8030) were purchased from Santa Cruz Biotechnology (Santa Cruz, CA). The CRM1 monoclonal antibody (1: 4000, 66763-1-Ig) was from ProteinTech (Wuhan, China). Anti-β-tubulin (1: 8000, HC101), anti-β-actin (1: 8000, HC201), anti-LaminA/C (anti-LamA/C, 1: 4000, HA105-01), anti-GST (1: 8000, HT601) and anti-His (1: 8000, HT501) mouse mAbs were purchased from TRANS (Beijing, China). Mouse mAb against PAR (1: 4000, ALX-804-220) was purchased from Alexis (San Diego, CA). Mouse mAb against FLAG (1: 8000, F1804) was purchased from Sigma-Aldrich (Saint Louis, MO). The anti-PAR rabbit polyclonal antibody (1: 4000, 4336-BPC-100) was from Trevigen (Gaithersburg, MD, USA). Anti-phosphoserine mouse polyclonal antibody (1: 4000, clone 4A4) was purchased from Merck Millipore (Billerica, MA, USA). Anti-p38 (1: 4000, 9212) and anti-phospho-p38 MAPK (Thr180/Tyr182, anti-p-p38, 1: 4000, 9211) rabbit polyclonal antibody were purchased from Cell Signaling Technology (Danvers, MA, USA).

### Cell culture, treatment, and transfection

Human Embryonic Kidney cells (HEK 293) was cultured in DMEM (Invitrogen) supplemented with 10% (v/v) Fetal Bovine Serum (FBS). The dose of recombinant human TNFα (Peprotech, 300-01A) was 10 ng/mL. Olaparib (Ola, AZD2281 Selleckchem, 10 nM), SB203580 (SB, RWJ 64809, MCE, 10 μM), Actinomycin D (Act D, M4881 AbMole, 10 μg/mL) and Cycloheximide (CHX, C1988 Sigma, 10 μg/mL) were added directly into the culture medium. siRNAs targeting PARP1 (5′-CCAAAGGAATTCCGAGAAA-3′) [22], sip38 (5′-GAAGCTCUCCAGACCATTT-3′) [16] and siCRM1 (5′-TGTGGTGAATTGCTTATAC-3′) [23], siHuR (5′-UGCCGUCACCAAUGUGAAAGU-3′) [8] were used at 100 nM. Lipofectamine 3000 (Invitrogen) and efficient transfection reagent (Abbkine) were used for transfection of siRNAs and plasmids.

### Constructs

To construct 3×Flag-p38, GST-p38 and His-p38 plasmid, the full-length of p38 was cloned into the vectors of pCMV-N-Flag, pGEX4T-2 and pET30a. Plasmids GST and GST-HuR were kindly provided by Dr. Myriam Gorospe (Laboratory of Cellular and Molecular Biology; National Institute on Aging, National Institutes of Health, USA). Plasmid GFP-HuR was provided by Dr. Imed-Eddine Gallouzi (Department of Biochemistry, Division of Critical Care, McGill University Health Center, McGill University, Montreal, Quebec H3G 146, Canada). His-HuR was amplified from GST-HuR plasmid and subcloned into His (pET30a) tagging vector between EcoRI and XhoI sites. A Fast Mutagenesis System kit (FM111, TRANS) was used to produce S197A, S197D, S197A-D226A and S197D-D226A mutations in His-HuR or GFP-HuR. The His-PARP1 was amplified by PCR using human cDNA as the template and was cloned into the pET28a vector. Mutagenesis System kit (FM111, TRANS) was used to produce GST-p38-T180D-Y182D (GST-p-p38) or PARP1-G972R, PARP1-Y986H and PARP1-Y986S site mutations in GST-p38 or His-PARP1.

### Cell fractionation

Total lysate, as well as cytoplasmic lysate and nuclear lysate were obtained as described [8, 9]. Briefly, cells were lysed in radioimmunoprecipitation assay (RIPA, W6001, US EVERBRIGHT) buffer for 30 min on ice. Lysate was centrifuged at 12,000×*g* for 20 min at 4 °C, and the supernatant was taken as the total lysate. Cytoplasmic and nuclear lysates were prepared by using the CelLytic NuCLEAR Extraction Kit (Sigma, NXTRACT, Saint Louis, MO). Briefly, cells were lysed with Cytosolic Lysis Buffer for 20 min, lysatee was centrifuged (11000×*g*, 1 min, 4 °C), and supernatant was collected as cytoplasmic lysate. The pellet was washed twice with Cytosolic Lysis Buffer and lysed with Extraction Buffer. Nuclear lysate was clarified by centrifugation (21,000×*g*, 5 min, 4 °C), and the supernatant was collected.

### Immunoblotting and Immunoprecipitation

Cells were cultured and stimulated as described above and lysed. For immunoblotting, 20–30 µg of proteins were separated by SDS polyacrylamide gels (SDS-PAGE) and transferred to nitrocellulose (NC) membrane, the membrane was blocked with 5% non-fat dry milk or 3% BSA (for phosphorylation) in TBST buffer (20 mM Tris base, 500 mM NaCl, 0.1% Tween-20, pH 7.5) for 1 h and incubated with the primary antibodies overnight at 4 °C. After 1 h incubation with horseradish peroxidase (HRP)-conjugated secondary antibodies, the protein signals were detected by using ECL plus a chemiluminescent detection system. For co-immuno-precipitation (Co-IP) analysis, 1×10^6^ cells were used, total lysate, as well as cytoplasmic lysate and nuclear lysate were incubated with antibodies recognizing HuR (3 μg), PARP1 (3 μg), GFP (3 μg) or FLAG (3 μg) over night at 4 °C. Protein G agarose beads were added into cell lysates and incubated at 4 °C for 3 h. Beads were washed three times with cell lysis buffer. Samples were separated by SDS-PAGE and subjected to immunoblotting with the indicated antibodies. Mouse IgG (Santa Cruz, CA) was used in control immuno-precipitation (IP) reactions. The relative band intensities were quantified by densitometry using the ImageJ software (1.41V, US National Institutes of Health).

### Immunofluorescence microscopy

Cells were seed into a 24-well plate and fixed with 10% (v/v) formaldehyde for 15 min and permeabilized with 0.5% (v/v) Triton X-100 for 10 min. The cells were washed with PBS and blocked with 2% (w/v) bovine serum albumin for 0.5 h at room temperature. After incubation with primary antibodies recognizing HuR (1:200), p38 (1:200), PARP1 (1:200) or FLAG (1:500) at 4 °C over night, the cells were subsequently incubated with Alexa Fluor 594/488-conjugated secondary antibodies for 1 h. The nucleus was stained with DAPI. After washing with PBST for three times, cells were observed and monitored by fluorescence microscopy (Zeiss).

To quantify the nuclear-cytoplasmic re-distribution of HuR and p38, densitometry analysis was conducted by using Image J software (version 1.44). The total HuR amount was measured first, and then that of nuclear HuR, thereby we had the cytoplasmic amount of HuR by taking nuclear amount of HuR away from that of the total. The re-distribution of HuR was estimated by dividing the cytoplasmic amount of HuR by that of total. The total p38 amount was measured first, and then that of nuclear p38. The re-distribution of p38 was estimated by dividing the nuclear amount of p38 by that of total.

### Duolink (proximity ligation assay, PLA)

The proximity ligation assay (PLA) (Dolin In Situ Detection Reagents Red, DUO92008, sigma) was performed following the manufacturer’s instructions. Cells growing on slides were fixed with paraformaldehyde 4% and blocked for 1 h, then incubated with the primary antibodies against the two kind of first antibodies overnight at 4 °C. The mouse and rabbit IgG were used as a negative control. A pair of oligonucleotide-labeled secondary antibodies (PLA probes) binds to the primary antibodies, and generates a signal only when the two probes are in proximity. Images were acquired on a confocal microscope (Zeiss).

### GST/His-fused protein purification and pull-down

Individual colonies of *E. coli* BL21 cells respectively transformed with plasmids carrying coding sequences for GST and GST/His-fused proteins were grown in LB medium (10 g/L tryptone, 5 g/L yeast extract and10 g/L NaCl) containing appropriate antibiotics for 12 h at 37 °C. The culture was grown to an optical density of 0.6–0.8 at 37 °C. After achieving the desired density, the induction was performed by adding 1 mM isopropyl- β-d-thiogalactopyranoside (IPTG) to an OD 1.0 culture at 37 °C for 3–4 h. Cells were harvested by centrifugation at 500 g and 4 °C for 10 min. The cell pellet was resuspended in bacterial lysis buffer (20 mM Hepes (pH 7.5), 120 mM NaCl, 10% v/v Glycerin, 2 mM EDTA and protease inhibitor). After breaking cell wall by ultrasonic and centrifugation at 13000 *g* for 25 min at 4 °C, the supernatant was used as the crude extract. The recombinant proteins were affinity-purified by glutathione Sepharose 4B (GE Healthcare Life Science, Uppsala, Sweden) or His-tag Purification Resin (P2221, Beyotime), and GST/His-tagged proteins were purified according to the manufacturer’s instructions. For pull-down experiments, GST and GST-fused proteins immobilized on 40 μL of Glutathione Sepharose 4B were incubated with 1 mL of purified His-fused proteins at 4 °C for 1–3 h. After three times washes with Nonidet P-40 lysis buffer, the bound proteins were analyzed by immunoblotting.

### In vitro assay of HuR phosphorylation and MS analysis for HuR phosphorylation

The compatible expression system for the production of highly phosphorylation recombinant protein in *Escherichia coli* was setted up [24–26]. Briefly, GST with His-HuR or GST-p-p38 (activated p38) with His-HuR were co-expressed in BL21 cells. A well-isolated single colony was selected and induced with 1 mM isopropyl-β-D-thiogalactopyranoside (IPTG) to an OD 1.0 culture at 37 °C for 3–4 h before lysis in bacterial lysis buffer (20 mM Hepes (pH 7.5), 120 mM NaCl, 10% v/v Glycerin, 2 mM EDTA and protease inhibitor) via sonication. Subsequently, the supernatant was purified with Ni-NTA agarose beads. The phosphorylation levels of HuR were analyzed by immunoblotting with an anti-pSer antibody.

For MS analysis for HuR phosphorylation, after staining proteins in SDS-PAGE gels with Coomassie brilliant blue, the gel slices in the position of His-HuR (40–45 kDa) were cut. The specific phosphorylation site (s) of His-HuR mediated by p38 was analysis by Shanghai Applied protein Technology (APT), China. Briefly, after the gel underwent destaining and fragmentation, a solution containing 20 ng/μL trypsin in 50 mM NH_4_HCO_3_ was added. Enzymatic digestion took place in a 37 °C incubator for 16 hours, followed by the extraction and freeze-drying of peptide extracts. Prior to analysis, the samples were resolubilized in a 0.1% formic acid solution and underwent peptide quantification before being subjected to mass spectrometry analysis. For the proteome profiling samples, peptides were analyzed on a Q Exactive HF-X Hybrid Quadrupole-Orbitrap Mass Spectrometer (Thermo Fisher Scientific) coupled with a high-performance liquid chromatography system (EASY nLC 1200, Thermo Fisher Scientific). Dried peptide samples re-dissolved in Solvent A (0.1% formic acid in water) were loaded onto a 2 cm self-packed trap column (100 μm inner diameter, 3 μm ReproSil-Pur C18-AQ beads, Dr. Maisch GmbH) using Solvent A and separated on a 150-μm-inner-diameter column with a length of 15 cm (1.9 μm ReproSil-Pur C18-AQ beads, Dr. Maisch GmbH) over a 150-min gradient (Solvent A: 0.1% formic acid in water; Solvent B: 0.1% formic acid in 80% ACN) at a constant flow rate of 600 nL/min (0–30 min, 0 min, 8% B; 0–4 min, 8–15% B; 4–19 min, 15–30% B; 19–22 min, 30–50% B; 22–23 min, 50–100% B; 23–30 min, 100% B). Eluted peptides were ionized at 2 kV and introduced into the mass spectrometer. Mass spectrometry was performed in data-dependent acquisition mode. For the MS1 Spectra full scan, ions with m/z ranging from 300 to 1400 were acquired by an Orbitrap mass analyzer at a high resolution of 120,000. The automatic gain control (AGC) target value was set to 3E+06. The maximal ion injection time was 80 ms. The top 60 precursor ions were selected for fragmentation in an HCD cell with a normalized collision energy of 27%. The resulting fragment ions were transferred to the Orbitrap analyzer, which operated at a resolution of 7500. The automatic gain control (AGC) was set to 5e4 for MS/MS. The maximum ion injection times were set to 20 ms. Dynamic exclusion of previously acquired precursor ions was enabled for 15 seconds. The original data of mass spectrometry analysis were RAW files, and iProteome one-stop data analysis cloud platform was used for qualitative and quantitative analysis.

### In vitro PARylation assay

A GST/His-fused protein in vitro PARylation assay was set up by modifying the method provided by the HT Universal Chemiluminescent PARP Assay kit (Trevigen, Gaithersburg, MD, USA). Briefly, GST and GST/His-fused proteins immobilized on glutathione Sepharose 4B were incubated with recombinant PARP enzyme (PARP-E) and PARP cocktail (buffer) with or without Ola at room temperature for 1 h. After three washes with Nonidet P-40 lysis buffer, the bound proteins were analysed by immunoblotting.

### PAR-binding assay ( dot-blot assay )

PAR was synthesized by using the in vitro PARylation system (HT Universal Colorimetric PARP Assay Kit) as described before [27]. Spotted PAR, GST and GST-fused proteins onto the nitrocellulose membranes after respectively diluted. The membrane was washed three times with TBST (10 mM Tris/HCl (pH 7.4), 150 mM NaCl, and 0.05% v/v Tween) and incubated in blocking buffer (20 mL 5% non-fat milk) for 30 min at room temperature. Subsequently, the membrane was incubated with 1 mL free PAR solution plus 1 mL PBS for 1 h at 37 °C. Anti-PAR mouse antibody (1: 4000) was used as the primary antibody and horseradish peroxidase-conjugated goat anti-mouse as the secondary antibody to probe the membrane, and the signal was detected with ECL reagent (S6010, US EVERBRIGHT).

### RNA Fluorescence in situ hybridization (RNA-Fish)

After various treatments, cells were fixed with 4% Paraformaldehyde (DingGuo, China) for 10 min at room temperature, permeabilized with 0.5% (v/v) Triton X-100 for 30 min, and then washed twice with 40% formamide for 10 min at room temperature. Thereafter, cells were pre-incubated with 1 mg/mL Salmon sperm DNA solution (10% Dextran sulfate sodium salt, 50% formamide, 2×SSC, 0.02% BSA, 2 mM VRC) at 42 °C for 1 h. After that, the cells were incubated with 20 ng/mL CXCL2-Cy5 probe overnight at 42 °C. The nuclei of the cells were stained with DAPI for 5 min. The sequence of the probe was Cy5-5’-CGGCTCCTGCGGGTGGCGCTGCTGCTCCTGCTCCTGGTGGCCGCC-3’ (Comate Bioscience Company, Changchun, China). Images were acquired on a confocal microscope (Zeiss).

### Reverse transcription and real-time PCR

Total RNA was extracted using TRIzol reagent (Invitrogen), and 1 μg of purified RNA from each sample was transcribed to cDNA. Primers for real time PCR included: TNFα (200 bp): forward: 5′- TCCAACCTTCCCAAACGC -3′, reverse: 5′- GTGGTTGCCAGCACTTCA -3′; CXCL1 (139 bp): forward: 5′- TCTCTCTTTCCTCTTCTGTTCCTA -3′, reverse: 5′- CATCCCCCATAGTTAAGAAAATCATC -3′; CXCL2 (149 bp): forward: 5′- CAAACCGAAGTCATAGCC -3′, reverse: 5′- GAACAGCCACCAATAAGC -3′; and β-actin (268 bp): forward: 5′-CTCCATCCTGGCCTCGCTGT -3′, reverse: 5′-GCTGTCACCTTCACCGTTCC -3′.

### Stability of mRNA

Endogenous HuR in HEK 293 cells were silenced using siRNA targeting HuR for 24 h, and GFP-HuR, GFP-HuR-S197A, GFP-HuR-S197D, GFP-HuR-D226A as well as GFP-HuR-S197D-D226A mutant plasmids were transfected. The plasmids were transfected into cells for 24 h, and then cells exposed to TNFα for 0.5 h, further with added transcription inhibitor Act D to the medium for 0 and 4 h. The level of mRNA was measured. Individual PCR amplification reactions were performed and analyzed as described previously [28].

### Statistical analysis

All experiments were performed at least three times for each determination. Data were expressed as means ± standard deviations (n≥3) and analyzed by one-way analyses of variance. The level of significance was accepted at * p < 0.05, ** p < 0.01 and *** p < 0.001.

## Results

### PARP1 and p38 are required for TNFα-induced HuR translocation from the nucleus to the cytoplasm

**Fig. 1:**
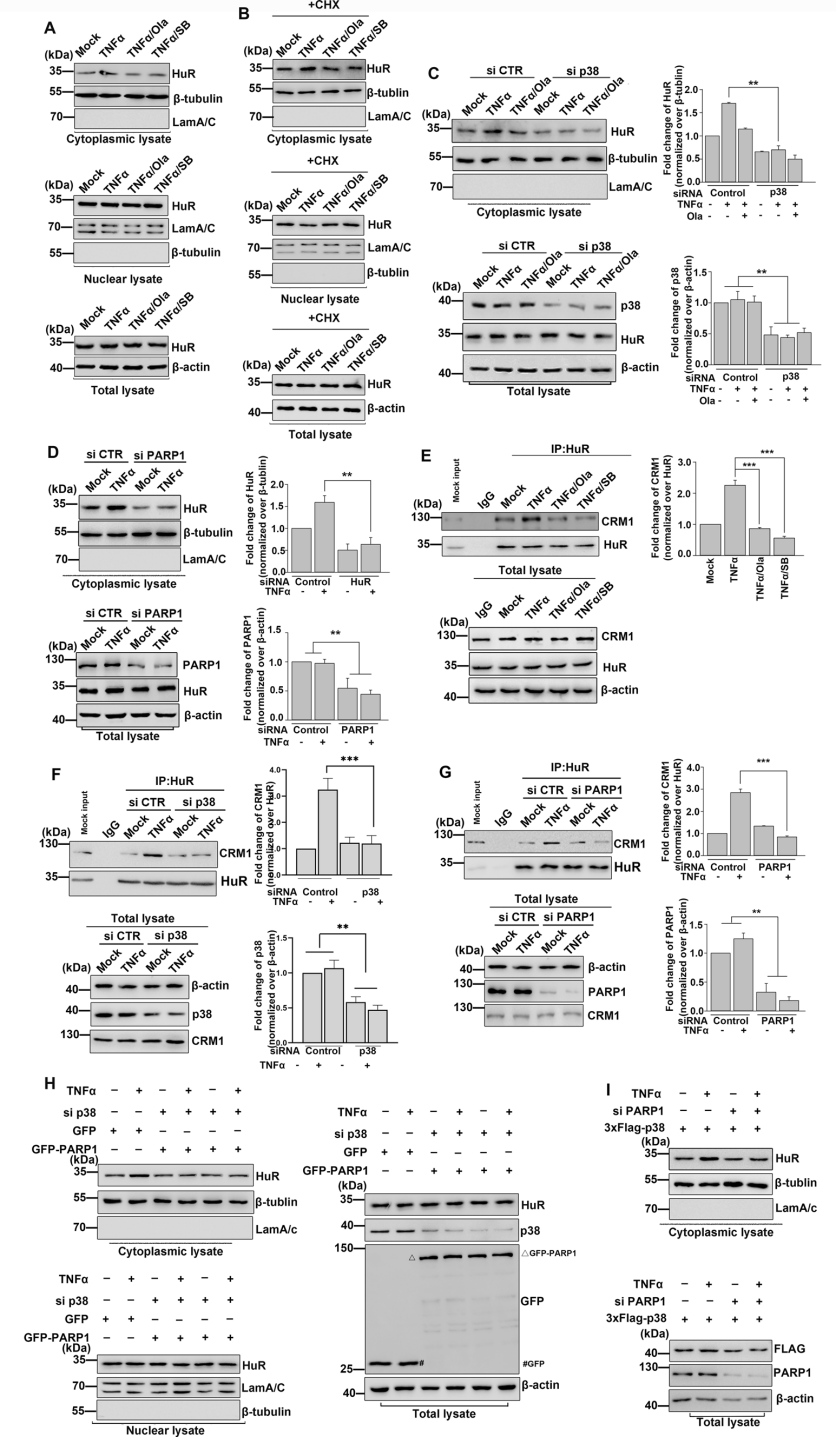
PARP1 and p38 increase the accumulation of HuR in cytoplasm **(A–D)** PARP1 and p38 knockdown decrease HuR translocation to the cytoplasm. **A** Cells were treated for 1 h with TNFα in the absence of Ola or SB. All inhibitors were pre-incubated for 10 min before the addition of TNFα. Cytoplasmic lysate (upper panel), nuclear lysate (middle panel) and total lysate (lower panel) were subjected to SDS-PAGE and immunoblotted with an anti-HuR specific antibody. Similar results were obtained from at least three independent experiments. **B** The increased cytoplasmic level of HuR is not due to its enhanced protein synthesis. HEK293 cells were exposed to cycloheximide (CHX) for 0.5 h and then added TNFα for 1 h. Cytoplasmic lysate (upper panel), nuclear lysate (middle panel) and total lysate (lower panel) were subjected to SDS-PAGE and immunoblotted with an anti-HuR specific antibody. **C, D** HEK293 cells were transfected with p38 siRNA **(C)**, PARP1 siRNA **(D)** or a control, and then, the cytoplasmic lysate (upper panel) and total lysate (lower panel) were subjected to SDS-PAGE and immunoblotted with an anti-HuR specific antibody. The results of immunoblotting were quantified by analysis of band densitometry using the ImageJ software. **p < 0.01 ; n=3. **E, F, G** Knockdown of PARP and p38 reduce the association of HuR and CRM1. **E** Cells were treated for 1 h with TNFα in the absence or presence of Ola or SB. Immunoprecipitates were prepared using an anti-HuR antibody and then subjected to immunoblotting analysis with the anti-CRM1 antibody. The results of immunoblotting were quantified by analysis of band densitometry using the ImageJ software. ***p < 0.001; n=3. **F, G** HEK293 cells were transfected with p38 siRNA (F), PARP1 siRNA (G) or a control, and then, cells were mock treated or TNFα exposed for 1 h. Immunoprecipitates were prepared using an anti-HuR antibody and then subjected to immunoblotting analysis with the anti-CRM1 antibody. The results of immunoblotting were quantified by analysis of band densitometry using the ImageJ software. **p < 0.01; ***p < 0.001; n=3 .**H, I** Both PARP1 and p38 are needed for the accumulation of HuR in cytoplasm. (H) HEK293 cells were transfected with p38 siRNA or a control, and then, cells were transfected with GFP or GFP-PARP1. After mock-treated or TNFα exposed for 1 h, cytoplasmic fractions, nuclear lysate and total lysate of cells were prepared and detected the localization of HuR by immunoblotting with an anti-HuR antibody. △, GFP-PARP1; #, GFP. (I) HEK293 cells were transfected with PARP1 siRNA or a control, and then cells were transfected with 3×Flag-p38. After mock-treated or TNFα exposed for 1 h, the cytoplasmic lysate (upper panel) and total lysate (lower panel) of cells were prepared and detected the shuttling of HuR by immunoblotting with an anti-HuR antibody

We first sought to determine whether PARP1 and p38 affected the localization of HuR in TNFα-treated cells by treating cells with the PARP/PARylation inhibitor Olaparib (Ola) and p38 inhibitor SB203580 (SB). We found that Ola and SB treatment led to a significant reduction in TNFα-induced accumulation of cytosolic HuR (Fig. 1A;S1A). To exclude the possibility that the increase in cytoplasmic HuR levels was due to enhanced protein synthesis, cells were also treated with the protein synthesis inhibitor cycloheximide (CHX). Our findings indicated that PARP1 and p38 activity were required for TNFα-induced HuR translocation from the nucleus to the cytoplasm independent of increased protein synthesis (Fig. 1B). Similar results were obtained following siRNA-mediated knockdown of PARP1 or p38 in cells (Fig. 1C, D; S1B). HuR has previously been shown to recruit CRM1 in the nucleus via adaptor proteins thereby allowing HuR to shuttle ARE-containing mRNAs to the cytoplasm [23]. We found that silencing CRM1 significantly blocked the export of HuR to the cytoplasm (Fig. S1C). Furthermore, HuR-CRM1 interactions were significantly reduced in the nuclei of Ola- or SB-treated cells compared with TNFα-treated cells (Fig. 1E), as well as in PARP1 or p38 knockdown cells (Fig. 1F, G). We next sought to confirm that both PARP1 and p38 were required for HuR translocation to the cytoplasm by transfecting GFP-PARP1 into endogenous p38-silenced cells and examining the cellular localization of HuR. We found that knockdown of p38 significantly blocked the TNFα-induced translocation of HuR to the cytoplasm, while overexpression of GFP-PARP1 did not increase the cytoplasmic accumulation of HuR in p38 knockdown cells (Fig. 1H). Moreover, overexpression of p38 led to increased HuR accumulation in the cytoplasm of TNFα-treated cells, while PARP1 knockdown resulted in a significant reduction in cytoplasmic HuR protein levels (Fig. 1I). Together, these findings suggest an indispensable role for PARP1 and p38 in regulating HuR cytoplasmic localization under inflammatory conditions.

### p38 MAPK mediates HuR phosphorylation at S197 following TNFα stimulation

**Fig. 2:**
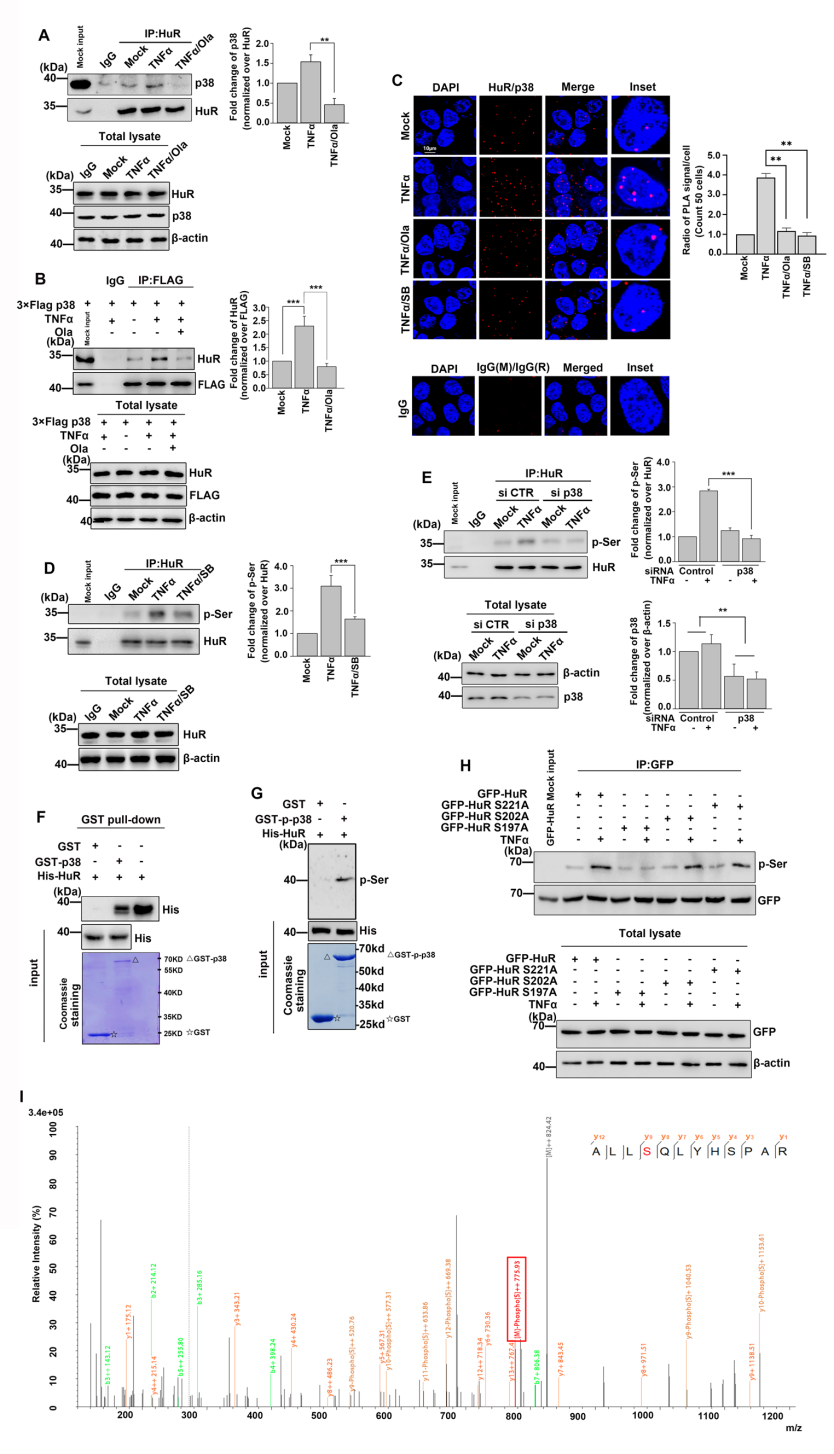
p38 binds with HuR and mediates the phosphorylation of HuR at S197 (A, B) TNFα increases the association of p38 with HuR. **A** HEK293 cells were either challenged with TNFα together with Ola or not for 1 h. Immunoprecipitates were prepared using an anti-HuR antibody and then subjected to immunoblotting analysis with the anti-p38 antibody. The results of immunoblotting were quantified by analysis of band densitometry using the ImageJ software. **p < 0.01; n=3. **B** HEK293 cells were transfected with 3×Flag-p38 plasmid, then cells were either challenged with TNFα together with Ola or not for 1 h. Immunoprecipitates were prepared using an anti-FLAG antibody and then subjected to immunoblotting analysis with the anti-HuR antibody. The results of immunoblotting were quantified by analysis of band densitometry using the ImageJ software. ***p < 0.001; n=3. **C** The interaction of p38 with HuR is analyzed by using a PLA assay. Cells were mock-treated or TNFα-exposed (± Ola/SB) for 1 h, and then subjected to PLA assays with indicated antibodies. Scale bar, 10 μm. The quantitative results are represented from three independent experiments. **p < 0.01. Scale bar, 10 μm. **D, E** p38 mediates the phosphorylation of HuR in response to TNFα exposure. HEK293 cells were either challenged with p38 inhibitor SB203580 **D** or transfected with p38 siRNA (E). Immunoprecipitates were prepared using an anti-HuR antibody and then subjected to immunoblotting to detect the phosphorylation of HuR.The results of immunoblotting were quantified by analysis of band densitometry using the ImageJ software. **p < 0.01. ***p < 0.001; n=3. **F** HuR directly interacts with p38. GST, GST-p38 and His-HuR were purified from bacteria and equal amounts of bead-coated GST and GST-p38 (visualized by coomassie brilliant blue staining, lower panel) were incubated with the same amount of soluble His-HuR (middle panel). The bound proteins were analyzed by immunoblotting with the anti-His antibody (upper panel). Similar results were obtained from at least three independent experiments. △, GST-p38; ☆, GST. **G** p38 mediated the phosphorylation of HuR in *E. coli*. GST with His-HuR or GST-p-p38 with His-HuR were co-expressed in *E. coli* BL21 cells, after a well-isolated single colony was selected and the expression of proteins were induced, His-HuR was purified with Ni-NTA agarose beads (middle panel). The phosphorylation level of HuR was analyzed by immunoblotting with an anti-pSer antibody. GST or GST-p-p38 protein was visualized by coomassie brilliant blue staining (lower panel). △, GST-p-p38; ☆, GST. **H** S197 site of HuR undergoes phosphorylation in TNFα-exposed cells. HEK293 cells were transfected with WT GFP-HuR as well as S197A, S202A and S221A mutant plasmids and then challenged with TNFα for 1 h or not. Immunoprecipitates were prepared using an anti-GFP antibody and then subjected to immunoblotting analysis to detect phosphorylation level of HuR. Similar results were obtained from at least three independent experiments. **I** Mass spectrometry validation of the S197 phosphorylation site in HuR. GST-p-p38 and His-HuR were co-expressed in *E. coli* BL21 cells, after a well-isolated single colony was selected and the expression of proteins were induced, His-HuR was purified with Ni-NTA agarose beads. Staining protein in SDS-PAGE gels with Coomassie brilliant blue, and then the excised gel slices were cut in the position of His-HuR (40-45kDa). Finally, the purified His-HuR proteins were trypsinized prior to MS analysis. The original data of mass spectrometry analysis were RAW files, and iProteome one-stop data analysis cloud platform was used for qualitative and quantitative analysis, which identified the peptides NVALLSQLYHSPAR. The y fragmentation was used to map the phosphorylation site to the Ser indicated in red.

We have previously shown that PARP1 interacts with HuR and PARylates HuR at D226 [8]. Here, our co-immunoprecipitation (IP) data revealed that TNFα stimulation led to increased interactions between HuR and p38 (Fig. 2A, B). In addition, our proximity ligation assay (PLA) showed a substantial increase in the interactions of p38 and HuR in TNFα-treated cells (Fig. 2C). Furthermore, we confirmed that HuR was phosphorylated by p38 using a phospho-Ser antibody. Treatment with a p38 inhibitor or knockdown of p38 resulted in a significant decrease in HuR phosphorylation in TNFα-stimulated cells (Fig. 2D, E).

To validate the direct interaction between p38 and HuR, we performed a pull-down assay by incubating GST and GST-p38 beads with purified His-HuR. The results showed that GST-p38 interacted with His-HuR under cell-free conditions, indicating that the two proteins interacted directly (Fig. 2F). Furthermore, the phosphorylation of p38 to HuR *in vitro* was examined by co-expressing GST-p-p38 (activated p38) and HuR in *Escherichia coli* as described in Materials and Methods [24–26]. The results revealed that the GST-p-p38 (GST-p38-T180D-T182D) and not GST induced phosphorylation of His-HuR, confirming that p38 mediates the phosphorylation of HuR directly (Fig. 2G).

Next, which site(s) of HuR might be phosphorylated was determined. Online software (GPS 5.0-Kinase-specific Phosphorylation Site Prediction (biocuckoo.cn)) predicted that Ser100 (S100), Thr118 (T118), Ser197 (S197), Ser202(S202) and Ser221(S221) residues on HuR were potential sites that might be phosphorylated by p38 (Fig. S2A). Since the HNS region or close proximity to the HNS region (spanning residues 205-237) is significant for HuR shuttling, we selected the S197, S202 and S221 sites on HuR to determine whether mutation of these sites could affect the phosphorylation of HuR under inflammatory conditions. Cells were transfected with wild type (WT) GFP-HuR as well as S197A, S202A and S221A mutant plasmids and then stimulated with TNFα. Immunoprecipitates were prepared using an anti-GFP antibody and then subjected to immunoblotting analysis to detect HuR phosphorylation levels. Mutation of S197 was consistently found to significantly block phosphorylation of HuR (Fig. 2H). Furthermore, mass spectrometry analysis revealed that GST-p-p38 (Fig. 2I), but not GST (Fig. S2B), mediated phosphorylation of HuR at the S197 site. Together, our findings suggest that p38 binds to HuR and phosphorylates HuR at its S197 site in response to TNFα treatment.

### PARylation of HuR at D226 facilitates its phosphorylation at S197

**Fig. 3:**
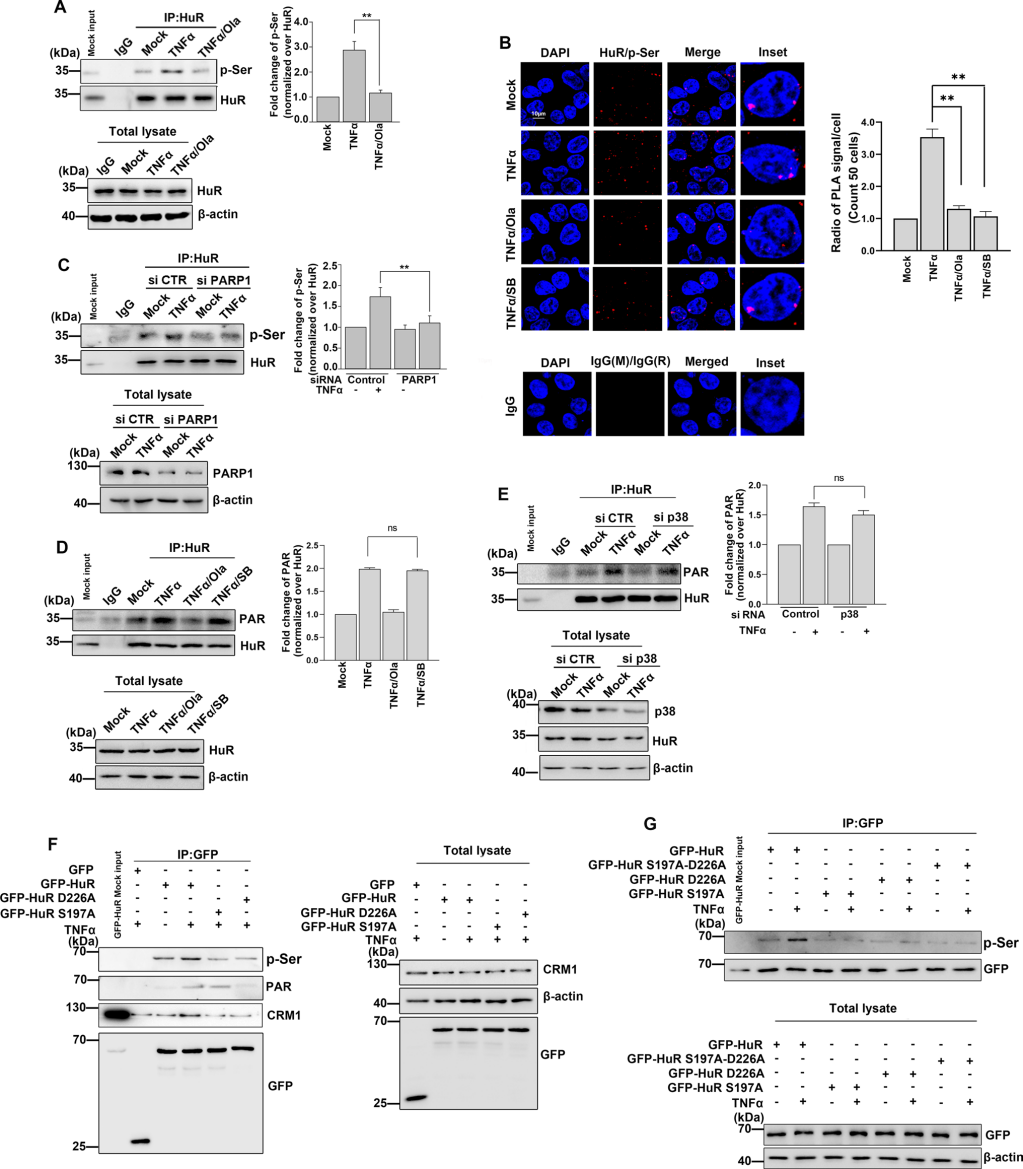
PARylation of HuR increases its phosphorylation **(A, B)** PARP inhibitor decreases the phosphorylation of HuR. **A** HEK293 cells were either challenged with TNFα together with Ola or not for 1 h. Immunoprecipitates were prepared using an anti-HuR antibody and then subjected to immunoblotting for the phosphorylation of HuR analysis. The results of immunoblotting were quantified by analysis of band densitometry using the ImageJ software. **p < 0.01; n=3. **B** The phosphorylation of HuR was analyzed by using a PLA assay. The cells were mock-treated or TNFα-exposed (± Ola/SB) for 1 h, and then subjected to PLA assays with indicated antibodies. The quantitative results are represented from three independent experiments. Scale bar, 10 μm. **p < 0.01. Scale bar, 10 μm. **C** PARP1 is required for the phosphorylation of HuR in response to TNFα stimulation. HEK293 cells were transfected with PARP1 siRNA or a control, then cells were mock-treated or TNFα-exposed for 1 h. Immunoprecipitates were prepared using an anti-HuR antibody, the phosphorylation of HuR was analyzed by immunoblotting with an anti-p-Ser antibody. The results of immunoblotting were quantified by analysis of band densitometry using the ImageJ software. **p < 0.01 ; n=3. **D, E** The activity of p38 dose not impact the PARylation of HuR. **D** HEK293 cells were either challenged with TNFα together with Ola/SB or not for 1 h. Immunoprecipitates were prepared using an anti-HuR antibody and then subjected to immunoblotting analysis for the PARylation of HuR. (E) HEK293 cells were transfected with p38 siRNA or a control, then cells were mock-treated or TNFα-exposed for 1 h. Immunoprecipitates were prepared using an anti-HuR antibody, the phosphorylation of HuR was analyzed by immunoblotting with an anti-PAR antibody. Similar results were obtained from at least three independent experiments. The results of immunoblotting were quantified by analysis of band densitometry using the ImageJ software. ns, no significant. **F, G** PARylation of HuR at D226 promotes its phosphorylation at S197. **F** HEK293 cells were transfected with WT GFP-HuR, S197A as well as D226A mutant plasmids and then challenged with TNFα for 1 h. Immunoprecipitates were prepared using an anti-GFP antibody and then subjected to immunoblotting analysis to detect the PARylation and phosphorylation level of HuR. Similar results were obtained from at least three independent experiments. HEK293 cells were transfected with GFP, GFP-HuR, GFP-HuR-S197A, GFP-HuR-D226A as well as GFP-HuR-S197A-D226A mutant plasmids and then challenged with TNFα for 1 h. Immunoprecipitates were prepared using an anti-GFP antibody and then subjected to immunoblotting analysis to detect the PARylation and phosphorylation level of HuR. Similar results were obtained from at least three independent experiments.

PARylation has previously been shown to act synergistically with other types of protein PTMs, such as phosphorylation, acetylation and ubiquitination [29–35]. Here, we found that HuR was PARylated and phosphorylated in response to TNFα stimulation, and that both modifications contributed to the accumulation of HuR in the cytoplasm. Thus, we next asked whether there was crosstalk between the PARylation of HuR and its phosphorylation, as well as how these two PTMs synergistically regulated HuR function. Using co-IP assays, we found that the increase in TNFα-induced HuR phosphorylation was significantly reduced in the presence of the PARP inhibitor, Ola (Fig. 3A). In addition, our PLA data revealed that Ola treatment reduced HuR phosphorylation following TNFα treatment (Fig. 3B). A similar phenomenon was observed through the knockdown of PARP1 (Fig. 3C). In addition, we found that inhibition or knockdown of p38 did not impair HuR PARylation (Fig 3D, E). We have previously shown that the D226 site on HuR is a major PARylation site. PARylation of D226 might lead to conformational changes in HuR, which facilitate interactions between HuR and its partners and/or mRNA targets, leading to the functional regulation of HuR [8–10]. Our findings here suggested that HuR PARylation was required for its phosphorylation. Thus, we examined the role of D226 in regulating HuR phosphorylation further by transfecting cells with WT GFP-HuR and D226A mutant plasmids, then stimulating them with TNFα. Immunoprecipitates were obtained using an anti-GFP antibody and HuR phosphorylation levels were detected by immunoblotting. We found significantly lower HuR phosphorylation levels in cells transfected with the D226A mutation plasmid than GFP-HuR transfected cells (Fig. 3F). In contrast, the mutation of HuR S197A did not affect the PARylation of HuR (Fig. 3F). In addition, cells were transfected with plasmids containing double mutations on both the D226 and S197 sites on HuR. Our IP data revealed that the S197A-D226A double mutation on HuR significantly reduced the phosphorylation of HuR similar to the effects of the S197A mutation alone (Fig. 3G). Together, our findings indicate that PARylation of HuR at D226 promotes its phosphorylation level at S197.

### PARylation is required for the nuclear localization of p38

**Fig. 4:**
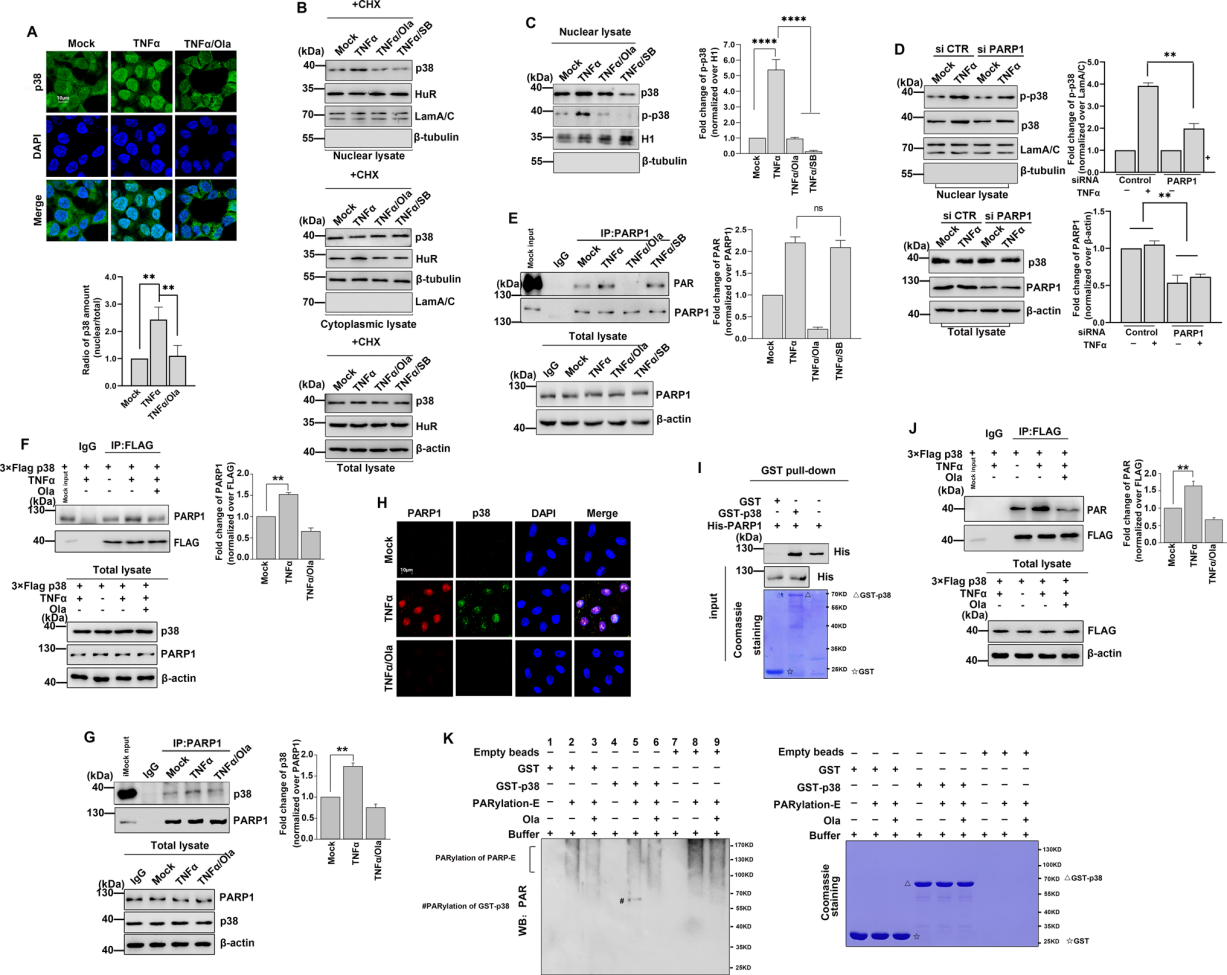
PARP1 is required for PARylation and activity of p38 under inflammatory factors stimulation **(A, B)** PARP inhibitor blocks the nuclear import of p38. **A** HEK293 cells were mock treated or TNFα treated with or without Ola for 1 h, then cells were fixed and permeabilized and incubated with anti-p38 antibody and FITC-conjugated secondary antibody. The nuclei of the cells were stained with DAPI. The nuclear distribution of p38 was quantified by densitometry analysis using Image J software (version 1.44) (lower panel) as described in Methods. ** p < 0.01. **B** The increased nuclear level of p38 is not due to its enhanced protein synthesis. HEK293 cells were exposed to cycloheximide (CHX) for 0.5 h and then added TNFα for 1 h. Cytoplasmic lysate (upper panel), nuclear lysate (middle panel) and total lysate (lower panel) were subjected to SDS-PAGE and immunoblotted with an anti-p38 specific antibody. **C, D** PARP increases the activity of p38. HEK293 cells were either challenged with TNFα together with SB or Ola for 1 h, the nuclear **(C)** lysates of different treated cells were subjected to SDS-PAGE and immunoblotted with anti-p38 and anti-p-p38 antibodies. Similar results were obtained from at least three independent experiments. **(D)** HEK293 cells were transfected with PARP siRNA or a control, and then, the phosphorylation of p38 was analyzed by immunoblotting with an anti-p-p38 antibody. Similar results were obtained from at least three independent experiments. The results of immunoblotting were quantified by analysis of band densitometry using the ImageJ software. **p < 0.01 ; n=3. **(E)** The activity of PARP1 is not influenced by p38. HEK293 cells were either challenged with TNFα together with Ola/SB or not for 1 h. Immunoprecipitates were prepared using an anti-PARP1 antibody and then subjected to immunoblotting analysis the PARylation of PARP1. Similar results were obtained from at least three independent experiments. The results of immunoblotting were quantified by analysis of band densitometry using the ImageJ software. ns, no significant; n=3.**(F), (G)** Exposure to TNFα agents increases the association of p38 with PARP1. HEK293 cells were transfected with 3×Flag-p38 plasmid (F) or not (G). HEK293 cells were mock treated or TNFα exposed (±Ola) for 1 h, then lysates from HEK293 cells were subjected to IP with anti-FLAG (F) or PARP1 (G) antibodies and immunoblotting analysis with the indicated antibodies. The results of immunoblotting were quantified by analysis of band densitometry using the ImageJ software. **p < 0.01; n=3. **H** Immunofluorescent staining of PARP1 and p38 in HEK293 cells. Cells were mock treated or TNFα exposed (±Ola) for 1 h, then cells were washed with CSK buffer (100 mM NaCl/300 mM sucrose/10 mM Pipes, pH 7.0/3 mM MgCl2, and protease inhibitors) plus 0.2%Triton X-100 to remove the soluble proteins, and then fixed. PARP1 (red) and p38 (green) were visualized by the indicated antibodies. Cells were counterstained with DAPI (blue). Scale bar, 10 μm. **I** p38 directly interacts with PARP1. GST, GST-p38 and His-PARP1 were purified from bacteria and equal amounts of bead-coated GST and GST-p38 (visualized by coomassie brilliant blue staining, lower panel) were incubated with the same amount of soluble His-PARP1 (middle panel). The bound proteins were analyzed by immunoblotting with the anti-His antibody (upper panel). Similar results were obtained from at least three independent experiments.△, GST-p38; ☆, GST. **J** TNFα exposure increases the PARylation level of p38. HEK293 cells were transfected with 3×Flag-p38 plasmid or not, then cells were either challenged with TNFα together with Ola or not. Immunoprecipitates were prepared using an anti-FLAG antibody and then subjected to immunoblotting analysis the PARylation of 3×Flag-p38. The results of immunoblotting were quantified by analysis of band densitometry using the ImageJ software. **p < 0.01; n=3. **K** p38 can be PARylated by PARP1 *in vitro*. Equal amounts of bead-coated GST, GST-p38 and empty beads (visualized by coomassie brilliant blue staining) were incubated with or without PARP-enzyme (PARP-E) in presence or absence of Ola, and then subjected to immunoblotting to detect PARylation levels. Similar results were obtained from at least three independent experiments. #, PARylation of GST-p38; △, GST-p38; ☆, GST.

Several studies have suggested that PARP1 may be involved in DNA damage repair and gene expression regulation by regulating p38, but the specific molecular mechanism remains unclear [21, 36–38]. To determine how PARP1 regulates p38 activation, we next examined the localization of p38 in response to TNFα treatment. Immunofluorescence staining revealed that p38 was predominantly localized in the cytoplasm of mock-treated cells. However, in TNFα-treated cells, nuclear translocation of p38 was observed, which was significantly inhibited by the addition of the PARP inhibitor (Fig. 4A). These differences in p38 were not due to altered protein stability, as shown by the treatment of TNFα-stimulated cells with CHX (Fig. 4B). Furthermore, the phosphorylation of p38 was found to be very weak in Ola-treated or PARP1 knockout cells compared with TNFα-treated cells (Fig. 4C, D). Finally, addition of the p38 inhibitor did not impair PARP1 activity (Fig. 4E and Fig. S3). Together, our results suggest that PARP1 may promote the phosphorylation of HuR by regulating p38 activity.

Next, we sought to examine the interactions between PARP1 and p38 using reciprocal IP assays. As shown in Fig. 4F, D, protein interactions between PARP1 and p38 were detected. In addition, we performed immunofluorescence staining to identify the interaction of p38 and PARP1 in cells by cytoskeleton (CSK) buffer treated. One hour after cells were treated with TNFα, soluble proteins were removed by pre-extraction with cytoskeleton (CSK) buffer that left only proteins associated with the cytoskeleton or chromatin. TNFα induced PARP1 activation and binding to chromatin, it is not extracted and the signal can be observed in our results (Fig. 4H), consistent with a previous study [39]. We also detected p38 after CSK buffer incubation (Fig. 4H), suggesting an interaction between p38 and PARP1. Furthermore, we found that His-PARP1 pulled down GST-p38 under cell-free conditions (Fig. 4I). Together, our findings suggest that PARP1 and p38 interact directly.

We next used IP assays to examine whether PARP1 covalently PARylated p38. Immunoprecipitation assay was performed, then probed the immunoprecipitation with an antibody against PAR. Results showed that p38 was PARylated, and that inhibition of PARP1 with a PARP inhibitor led to a reduction in p38 PARylation levels (Fig. 4J). Using an *in vitro* PARylation assay as described in the Materials and Methods, we found that incubation with the PARP enzyme resulted in strong modifications of GST-p38 (Fig. 4K, compare Lanes 4–5), but not GST (Fig. 4K, Lanes 1–3). Reduced p38 modification was observed in the presence of Ola (Fig. 4K, Lane 6). Taken together, our results suggest that PARP1 can mediate the PARylation of p38 and promote the activation of p38.

### p38 is a new PAR-binding protein that binds preferentially to moderately hyperbranched PAR chains

**Fig. 5:**
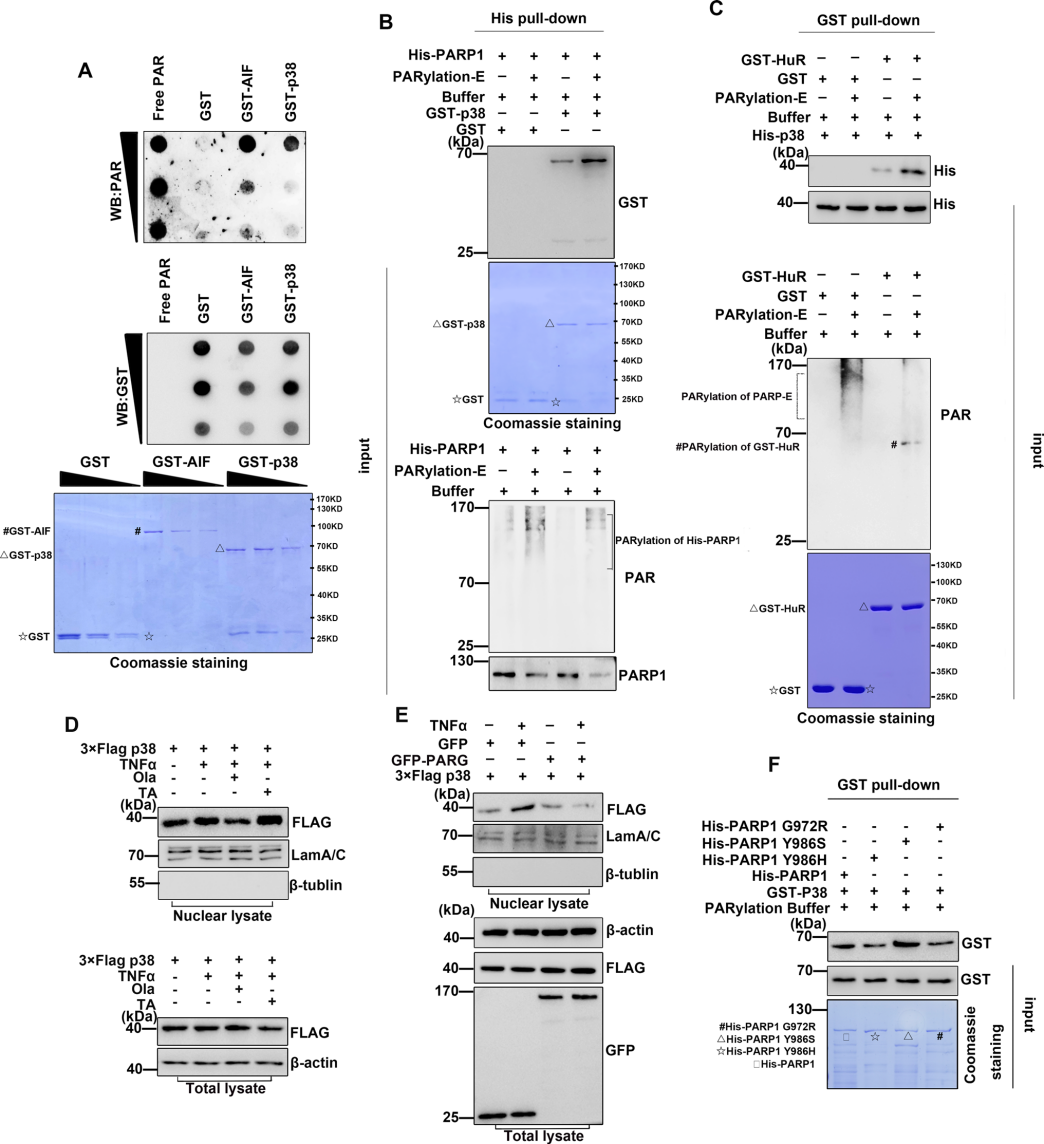
The p38 is a new PAR-binding protein that binds with moderately hyper PAR chain **(A)** The p38 is a new PAR-binding protein. Purified GST (negative control), GST-AIF (positive control), GST-p38 recombinant proteins were spotted on the nitrocellulose membrane respectively, and incubated with free PAR, immunoblotting was performed to detect the bound PAR levels. Similar results were obtained from at least three independent experiments. **B, C **The combination of PAR and p38 increases the interaction of p38 with PARP1 and HuR. **B** Auto PARylation of bead-coated His-PARP1 was performed or not (lower panel) and then incubated with equal amounts of soluble GST or GST-p38 (visualized by coomassie brilliant blue staining, middle panel), the bound proteins were analyzed by immunoblotting with the anti-GST antibody (upper panel). △, GST-p38; ☆, GST. **C** Equal amounts of bead-coated GST and GST-HuR (visualized by coomassie brilliant blue staining, lower panel) were incubated with or without PARP-enzyme (PARP-E) (middle panel), then incubated with equal amounts of soluble His-p38, the bound proteins were analyzed by immunoblotting with the anti-His antibody (upper panel). #, PARylation of GST-HuR; △, GST-HuR; ☆, GST. **D, E** PARG reduces the activity of p38. HEK293 cells were treated with PARG inhibitor TA **(D)** or transfected with GFP-PARPG or GFP **(E)** after transfected with 3×Flag-p38 plasmid, the localization of 3×Flag-p38 were analysis by immunoblotting staining with an anti-FLAG antibody. **F** The p38 prefers to bind short and moderately hyper branched PAR chains. Auto PARylation of different PARP1 variants were performed (visualized by coomassie brilliant blue staining, lower panel), and then incubated with bead-coated GST-p38 protein (middle panel). The interaction of different PAR chains with p38 was detected with the anti-GST antibody (upper panel).

Interestingly, inhibiting PARP1 activity with the PARP inhibitor significantly reduced the interactions between p38 and HuR or PARP1 (Figs. 2A, B; 4F, G), suggesting that the PAR chain may be a pre-requisite for p38-PARP1/HuR interactions. Recent studies have demonstrated that reading the PAR signal by PAR-binding proteins through non-covalent bonds constitutes a major aspect of PAR biology, and that many acceptors of covalent PARylation also read PAR signals [40–46]. We assessed the PAR-binding properties of p38 using a dot-blot assay [47], with GST as a negative control and apoptosis inducing factor (AIF) as a positive control [48]. Here, we found that p38 displayed a strong affinity for PAR (Fig. 5A). Next, we asked whether PAR chains were sufficient to promote the interactions between p38 and PARP1 or HuR. GST, GST-HuR or His-PARP1 were purified and subjected to PARylation or not, then incubated with His-p38 or GST and GST-p38, respectively. We found that recombinant p38 significantly combined with PARylated HuR or PARP1 compared with the unmodified proteins (Fig. 5B, C). Following activation of PARP1, poly (ADP-ribose) glycohydrolase (PARG) is immediately activated and hydrolyzes the PAR chain on the target protein. Here, we found using immunofluorescence staining that treatment with the PARG inhibitor tannic acid (TA) led to a significant increase in the nuclear localization of 3×Flag-p38 compared to TNFα-treated cells (Fig. 5D). In contrast, overexpression of GFP-PARG inhibited the accumulation of 3×Flag-p38 in the nucleus, further highlighting an important role for PAR chains in the nuclear accumulation of p38 (Fig. 5E).

To obtain further insight into the role of the structural heterogeneity of PAR regarding chain length and branching in cellular (patho-) physiology, we used different PARP1 variants that have been previously reported to synthesize PAR with different properties [49]. The variant PARP1-G972R is known to produce short and hyperbranched PAR, while the variant PARP1-Y986H produces strongly hyperbranched PAR, and PARP1-Y986S produces short and moderately hyperbranched PAR. We purified the respective proteins using an *E. coli* expression system (Fig. S3A), and analyzed the PAR produced (Fig. S3B) [49]. Following auto-PARylation of PARP1 or its mutations, we performed pull down assays with GST-p38 protein. We found that PARylation of WT PARP1 and its mutants displayed increased p38 binding with the strongest binding for PARP1-Y986S (Fig. 5F). Taken together, our results suggest that p38 is a PAR binding protein that binds preferentially to short and moderately hyperbranched PAR.

### The PARP1-p38 *axis* is critical for HuR cytoplasmic accumulation and inflammatory gene expression in TNFα-treated cells

**Fig. 6:**
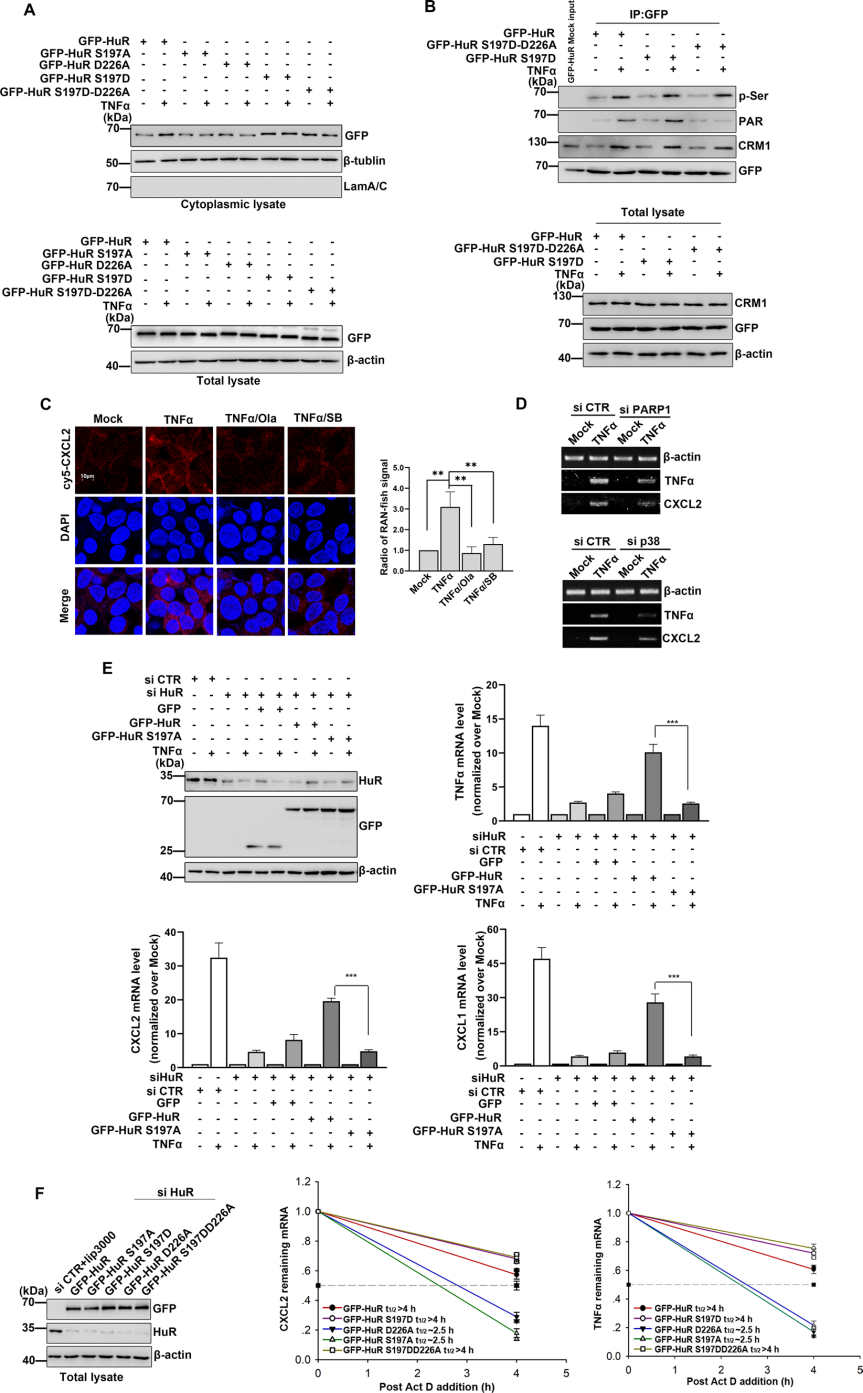
PARP1 and p38 synergistically regulate inflammatory gene expression **(A, B)** The phosphorylation of HuR S197 is necessary for HuR cytoplasmic localization. (A) HEK293 cells were transfected with GFP-HuR, GFP-HuR-S197A, GFP-HuR-D226A, GFP-HuR-S197D, GFP-HuR-S197D-D226A plasmids. After mock-treated or TNFα exposed for 1 h. Cytoplasmic lysate (upper panel) and total lysate (lower panel) cells were prepared, and HuR levels in the cytoplasm were detected by immunoblotting with anti-GFP antibody. **B** HEK293 cells were transfected with GFP-HuR, GFP-HuR-S197D, GFP-HuR-S197D-D226A plasmids. After mock-treated or TNFα exposed for 1 h. Immunoprecipitation was prepared by using an anti-GFP antibody and then subjected to immunoblotting analysis with the anti-PARylation, anti-phosphorylation and anti-CRM1 antibodies. Similar results were obtained from at least three independent experiments. **C** Analysis of the expression of CXCL2 in PARP inhibitor Ola or p38 inhibitor SB203580 treated cells using a RNA FISH assay. Cells were treated with TNFα for 1 h in the absence or presence of Ola or SB.The levels of CXCL2 mRNA were determined by fluorescence *in situ* hybridization (red). The nuclei of cells were counter stained with DAPI. Bar,10 μm. The quantitative results are represented from three independent experiments.**p < 0.01. **D** PARP1 and p38 knockdown block the upregulation of inflammation related genes. HEK293 cells were subjected to siRNA targeting PARP1 or p38 and then mock treated or exposed to TNFα for 1 h; RT- PCR was performed to detect the mRNA expression of TNFa and CXCL2. A representative result of three independent experiments is shown. **E, F** S197 site mutation of HuR inhibites the stability of inflammatory gene. **E** Inflammation related mRNA expression is impaired in S197A HuR-expressing cells. Endogenous HuR in HEK 293 cells was silenced using siRNA targeting a distinguished sequence of HuR, and then GFP, WT GFP-HuR and GFP-S197A HuR plasmids were transfected. Immunoblotting was performed to detect the interference of endogenous HuR, as well as the ectopic expression of GFP-tagged murine WT and S197A-HuR . Cells expressing WT or S197A HuR were stimulated with TNFα, or not for 1 h. TNFα , mRNA levels of CXCL1 and CXCL2 were detected by real-time PCR. Data were expressed as mean ± SD (n=3), and analyzed by one-way ANOVA. ***p < 0.001. (F) Endogenous HuR in HEK 293 cells were silenced using siRNA targeting HuR for 24 h, GFP-HuR, GFP-HuR-S197A, GFP-HuR-S197D, GFP-HuR-D226A as well as GFP-HuR-S197D-D226A mutant plasmids were transfected (left). The plasmids were transfected into cells for 24h, and then exposed to TNFαfor 0.5 h, further with added transcription inhibitor Act D to the medium for 0 and 4 h. The level of mRNA was detected by real-time PCR.

To gain further insight into the pathophysiological significance of HuR D226 PARylation and HuR S197 phosphorylation in an intracellular context, we next transfected cells with WT GFP-HuR, GFP-HuR-S197A, GFP-HuR-D226A, GFP-HuR-S197D and GFP-HuR-S197D-D226A plasmids and examined the localization of HuR. We found that the D226 mutation significantly blocked accumulation of HuR in the cytoplasm. Mutation of S197 to non-phosphorylated (S197A) prevented the cytoplasmic translocation of HuR even though HuR was PARylated, while phosphorylation of S197 (S197D) significantly promoted HuR cytoplasmic accumulation even without PARylation of HuR (Fig. 6A, B). These findings suggest that PARylation alone is insufficient for the translocation of HuR, while S197 phosphorylation of HuR is necessary for the cytoplasmic accumulation of HuR.

Our RNA fluorescence in situ hybridization (FISH) data revealed that treatment with the PARP1 inhibitor or p38 inhibitor significantly inhibited the expression of pro-inflammatory genes (Fig. 6C). These results were consistent with our RT-PCR analysis, which showed a significant reduction in the levels of pro-inflammatory genes, such as CXCL2 and TNFα in PARP1- or p38-depleted cells (Fig. 6D). Silencing endogenous HuR in HEK 293 cells strongly blocked TNFα-induced increases in inflammatory-associated mRNA levels, which were markedly rescued by the overexpression of WT HuR but not S197A HuR (Fig. 6E). The stability of inflammatory-associated mRNAs was further determined by transfecting endogenous HuR-silenced HEK 293 cells with GFP-HuR, GFP-HuR-S197A, GFP-HuR-S197D, GFP-HuR-D226A and GFP-HuR-S197D-D226A mutant plasmids. We found that the half-lives of inflammatory mRNAs in GFP-HuR-expressing cells exceeded 4 h compared to approximately 2.5 h in S197A or D226A HuR-expressing cells. Mutation of S197 into phosphorylated S197D led to the restoration of the inflammatory cytokine half-lives to levels similar to those observed in GFP-HuR-expressing cells (Fig. 6F). Taken together, these results indicate that phosphorylation of HuR at S197 can promote HuR accumulation in the cytoplasm, thus promoting the stability and expression of inflammatory genes.

## Discussion

**Fig. 7:**
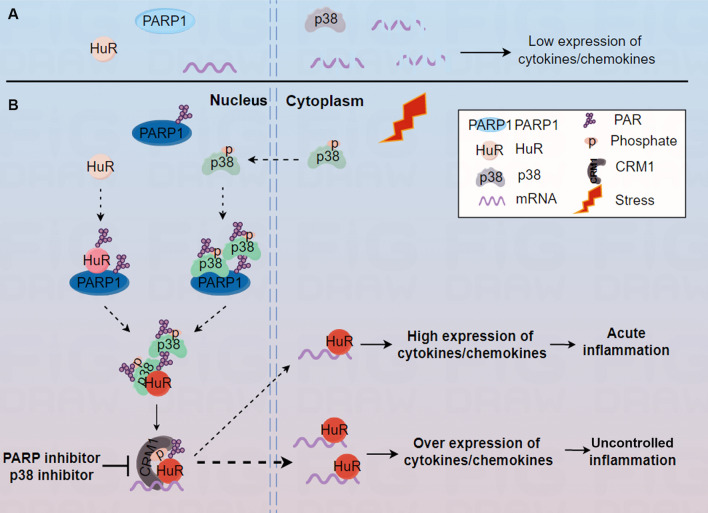
Potential models of PARP1-p38 axis in regulating gene expression by enhancing cytoplasm accumulation of HuR **(A)** Cells maintain low expression of cytokine/chemokine mRNA in normal state. **B** Cells overexpress cytokine/chemokine mRNA in the inflammatory state. Upon cell is under inflammation stimulation, PARP1 is activated and PARylates itself, as well as HuR. At the same time, the PAR chain recruits p38 and then PARylated p38, which promoted the nuclear localization and activity of p38. Moreover, the PAR chains of HuR at D226 increased the binding of p38 to HuR, which further phosphorylated HuR at S197 site. Eventually the phosphorylated HuR is more easier recognised by CRM1 and then transports mRNA into the cytoplasm.

Dysregulated immune responses caused by dysfunctional transcriptional or post-transcriptional regulation can lead to inflammatory diseases or cancer [50]. Both PARP1 and p38 are well-defined stress-activated enzymes involved in many inflammatory diseases [10, 51–53]. To date, the majority of studies on PARP1 and p38 have focused on the transcriptional control of genes. However, post-transcriptional regulation of mRNA in eukaryotes is equally important but understudied. In this study, we uncover a functional and mechanistic link between PARP1 and p38 in the regulation of the RNA binding protein HuR, which regulates subcellular localization and inflammatory gene stability at the post-transcriptional level in response to inflammatory factor stimulation (Fig. 7).

PARP1- and p38-mediated HuR PTMs play crucial roles in the regulation of HuR subcellular localization and subsequent mRNA stability under inflammatory conditions. HuR interacts with approximately 15% of the cellular transcriptome [54], and has been identified as a key regulator of post-transcriptional mRNA metabolism in the cytoplasm [55, 56]. HuR translocation to the cytoplasm is governed by PTMs mediated by different kinases [1, 8, 15, 23, 57]. Here, we show that blocking PARP or p38 activity significantly reduced the cytoplasmic accumulation of HuR (Fig. 1). Previously, we have shown that PARP1 interacts with HuR and mediates PARylation of HuR at its D226 site [8]. Here, we confirm that p38 binds to and mediates the phosphorylation of HuR (Fig. 2). Online software predicted that the S197, S202 and S221 sites in the HuR HNS region are potential sites for HuR phosphorylation. Previous studies have reported that phosphorylation of HuR by CDK1 at S202 retains HuR in the nucleus [58]. Moreover, PKCα has been shown to interact with HuR in the nucleus to phosphorylate HuR S221, leading to ATP-dependent HuR cytoplasmic translocation [59]. In this study, we identify S197 on HuR as a novel and important phosphorylation site for p38 phosphorylation in response to TNFα stimulation. More importantly, cytoplasmic accumulation of HuR and the stability of inflammatory-associated mRNAs were reduced in S197A or D226A mutated HuR compared to WT HuR (Figs. 2, 6).

PARylation of the D226 site on HuR promotes phosphorylation of HuR at S197. Interestingly, we found that TNFα treatment did not lead to HuR cytoplasmic migration in p38 knockout cells overexpressing PARP1 or in PARP1 knockout cells overexpressing p38, suggesting that PARP1 and p38 may act synergistically to regulate HuR localization. PARylation has been found to crosstalk with many other types of protein PTMs (termed 'PAR-dependent PTMs') including phosphorylation, acetylation and ubiquitination [29–33]. PARP family members are thought to PARylate a substrate protein, which promotes PAR-dependent interactions with a modifying enzyme, leading to PTM of the PARP substrate protein by the modifying enzyme [35]. For example, PARP1-dependent PARylation of STAT1a at the A721 site has been shown to promote subsequent interferon gamma-mediated transactivation through phosphorylation at S727 in macrophages [34]. In this study, we show that HuR phosphorylation is significantly attenuated in TNFα-stimulated cells treated with the PARP inhibitor or after PARP1 interference. In contrast, blocking the activity of p38 does not affect TNFα-induced PARylation of HuR. Furthermore, mutating the D226 site also significantly inhibits HuR phosphorylation, similar to the effects of HuR S197A mutation. The phosphorylation of HuR is also inhibited by D226 and S197 double mutation (Fig. 3). Mutation of HuR S197 into the phosphorylated state (S197D) results in significantly increased translocation of HuR from the nucleus to the cytoplasm, even without PARylation of HuR (Fig. 6). These results suggest that the p38-dependent phosphorylation of HuR at S197 is readily recognized by CRM1, and necessary for HuR accumulation in the cytoplasm.

PARylation is required for the activity and nuclear localization of p38. PARP1 and PARylation have been found to be involved in the activation and cellular localization of multiple kinases. For example, PARP1 has been shown to PARylate RelA/p65 and induce RelA/p65 nuclear retention and transcriptional function [60]. Under DNA damaging conditions, super-activated PARP1 was found to induce PARylation of p53, then promote the accumulation and transcriptional function of p53 in the nucleus [61]. Here, we identify p38 as a novel binding partner and PARylation target of PARP1 (Fig. 4). Nuclear import of p38 and p38 phosphorylation levels are significantly increased in TNFα-treated cells, but reduced in the presence of PARP inhibitors or following PARP1 knockdown (Fig. 4). Under inflammatory conditions, p38 protein has been shown to translocate to the nucleus following activation of the MAPK signaling pathway via auto-phosphorylation and binding to nuclear importins (Imp7/3 or Imp9/3 dimers), where it participates in gene expression [51, 62]. Our results confirm the important role of PARylation in p38 activation and nuclear localization. However, further studies are required to elucidate the mechanism of crosstalk between p38 phosphorylation and PARylation in response to TNFα stimulation.

The MAPK p38 is a new PAR reader, which can non-covalently bind with moderately hyperbranched PAR chains. As a cellular sensor, PARP1 can be activated by various stress stimuli with the levels of PARP1 activity and PAR synthesis increasing as the strength of the stress stimuli increases. PAR chains of specific lengths and branching frequencies are thought to exert specific functions, called the ‘PAR code’ [49]. In addition to proteins covalently PARylated by PARP family members, non-covalent PAR-binding proteins constitute another major aspect of PAR biology [41, 44, 63]. Histones, CHK1, p53 or XPA preferentially bind to PAR chains of specific lengths [30, 31, 41, 64–66]. Interestingly, PAR binding proteins are also the acceptors of covalent PARylation, which is known as 'PAR-dependent PARylation' [35, 64]. Here, we show that p38 non-covalently binds to PAR chains, and is more likely to bind to short and moderately hyperbranched PAR chains (Fig. 5). Moreover, we show that anto-PARylated or PARylated HuR recruits more p38 (Fig. 5). Although we do not know the nature of the stimulating activity by short and moderately hyperbranched chains of PAR, PARP1 is reportedly more likely to form short and moderately hyperbranched PAR chains under inflammatory conditions. Furthermore, this kind of chain or high local concentration of PAR acts as a scaffold that places p38 in the spatial proximity of the catalytic center of PARP1 leading to covalent PARylation of p38 by PARP1.

In summary, our study identifies a novel mechanism for the PARP1-p38 axis in promoting the stability of inflammatory genes by altering the localization of the RNA-binding protein HuR in cells. This study advances our understanding of the molecular mechanisms through which multiple signaling pathways or enzymes in cells work together to fine-tune the expression of inflammatory genes at the post-transcriptional level. Moreover, our findings highlight the significance of PARP or p38 inhibitors in the treatment of uncontrolled inflammation and some cancers (Fig. 7).

### Supplementary Information

Below is the link to the electronic supplementary material.

Supplementary file1 (DOCX 1945 KB)

Supplementary file2 (PDF 1351 KB)

## Data Availability

The data supporting the results of this article are provided in the on-line Supplementary Datafle 1.

## Abbreviations

AREs: AU-rich elements
RBPs: RNA-binding proteins (RBPs)
HNS: Nucleocytoplasmic shuttling sequence
ELAVL: Embryonic lethal abnormal vision-like
PTM: Post-translational modification
PARP1: Poly (ADP-ribose) polymerase 1
PARylation: Poly ADP-ribosylation
PAR: Poly ADP-ribose
MAPK: Mitogen-activated protein kinase
HEK293: Human Embryonic Kidney cells
Ola: Olaparib
Act D: Actinomycin D
SB: SB203580
PLA: Proximity ligation assay
WT: Wild-type
