# Supplementary material for: Poly (ADP-ribose) polymerase 1 promotes HuR/ELAVL1 cytoplasmic localization and inflammatory gene expression by regulating p38 MAPK activity
Source: Cell Mol Life Sci. 2024 Jun 9;81(1):253. doi: 10.1007/s00018-024-05292-2 (PMC11335290; doi:10.1007/s00018-024-05292-2)
Supplement: Supplementary file 1 — Supplementary file1 (DOCX 1945 KB) [file 18_2024_5292_MOESM1_ESM.docx]

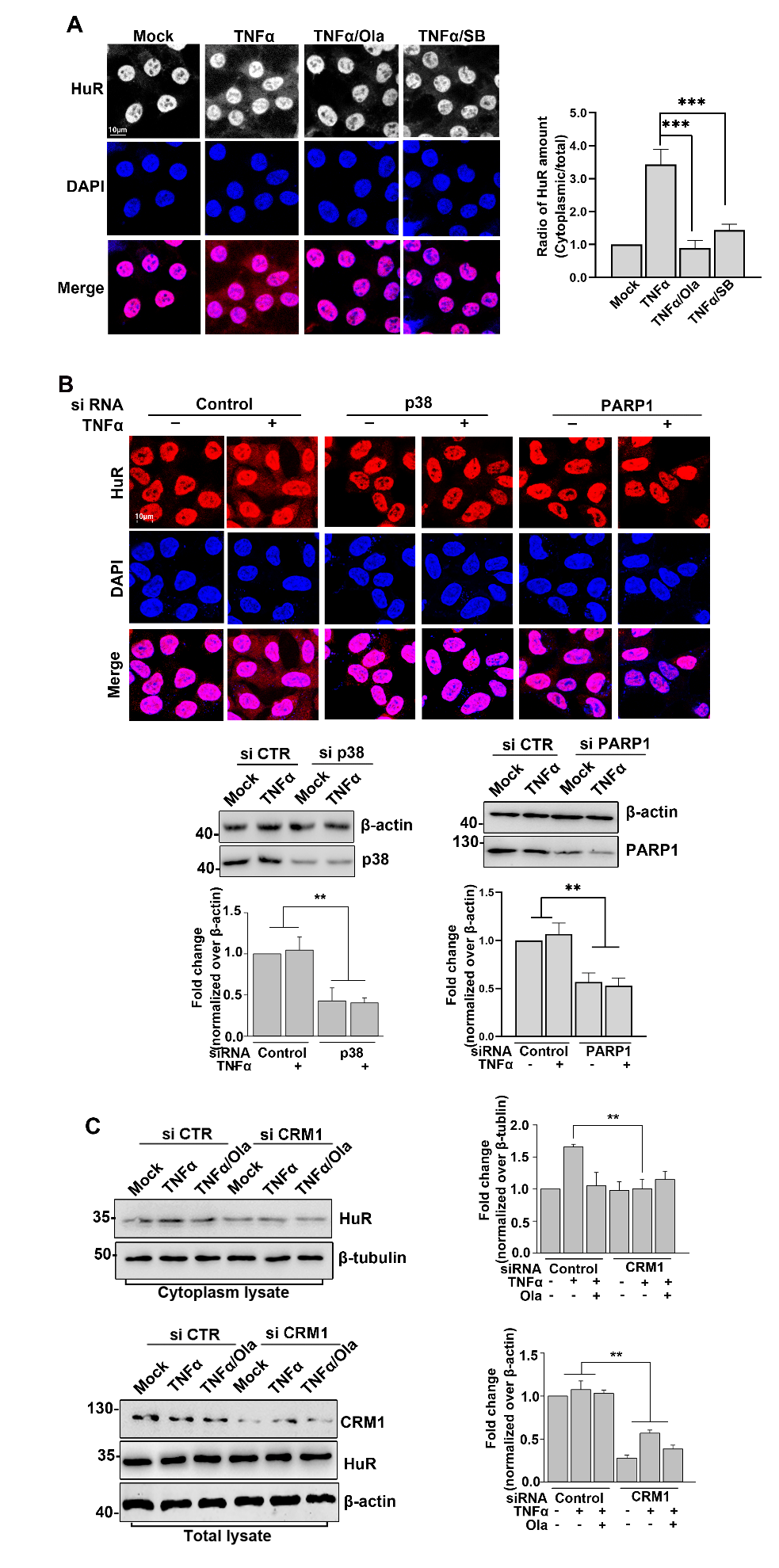


**Fig.S1 PARP1 and p38 are both required for HuR accumulation in the cytoplasm**

**(A)** PARP1 and p38 increase the accumulation of HuR in cytoplasm. HEK293 cells were treated for 1 h with TNFα in the absence of Ola or SB, and then immune-fluorescence (IF) stained with an anti-HuR antibody. The cytoplasmic distribution of HuR was quantified by densitometry analysis using Image J software (version 1.44) (Right panel) as described in Methods.*** p < 0.001.

**(B)** PARP1 and p38 knockdown decrease HuR localization in the cytoplasm. HEK293 cells were transfected with p38 siRNA, PARP1 siRNA or a control, and then immune-fluorescence (IF) stained with an anti-HuR antibody. The results of immunoblotting were quantified by analysis of band densitometry using the ImageJ software. **p < 0.01 ; ***p < 0.001; n＝3. Scale bar, 10 μm.

**(C)** CRM1 mediates the shuttling of HuR from nucleus to cytoplasm in TNFα treated cells. HEK293 cells were transfected with CRM1 siRNA or a control, and then, the cytoplasm localization of HuR was analyzed by immunoblotting with an anti-HuR antibody. The results of immunoblotting were quantified by analysis of band densitometry using the ImageJ software. **p < 0.01; n＝3.


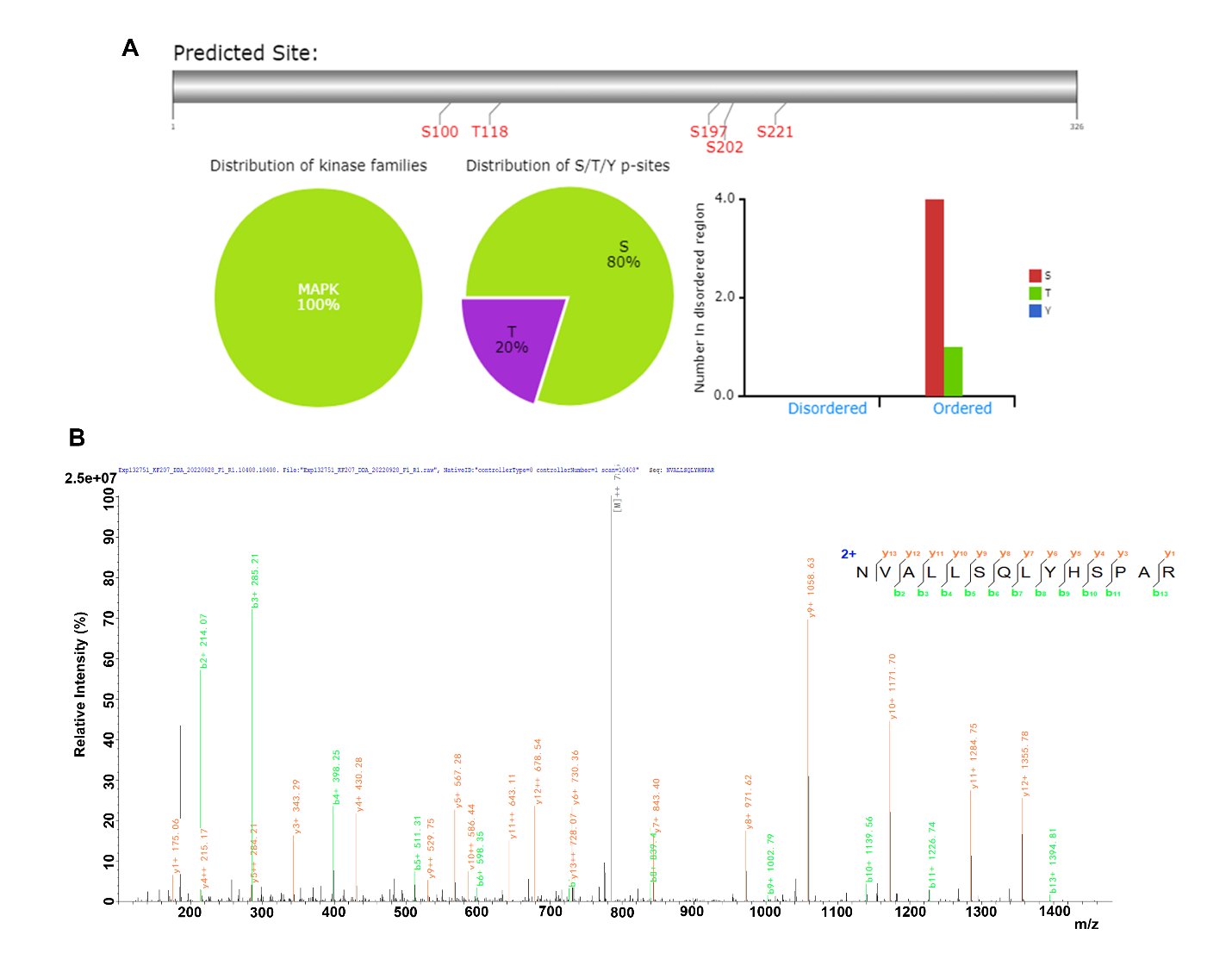


**Fig. S2 HuR was phosphorylated by p38 at S197 under TNFα treatment**

**(A)** Online software (http://www.csspalm.biocuckoo.org/online.php) predictes that Thr118 (T118), Ser197 (S197), Ser 202 (S202), and Ser 221 (S221) are potential sites of phosphorylation for HuR by p38.

**(B)** Protein mass spectrometry assay. Protein mass spectrometry assay. GST and His-HuR were expressed in *E. coli* BL21 cells, and then His-HuR was purified with Ni-NTA agarose beads. After staining protein in SDS-PAGE gels with Coomassie brilliant blue, the excised gel slices were cut in the position of His-HuR (40-45kDa) and then the purified His-HuR proteins were trypsinized prior to MS analysis.


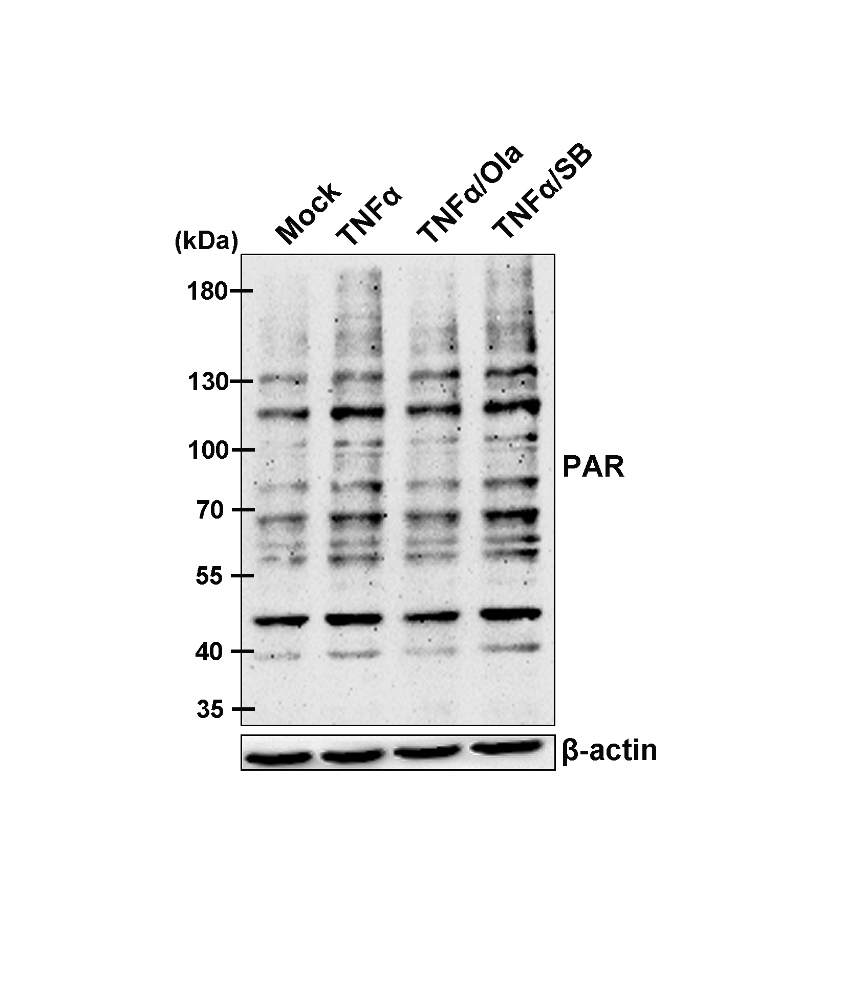


**Fig.S3 The activity of PARP1 is not influenced by p38**

HEK293 cells were either challenged with TNFα together with Ola/SB or not for 1 h. Protein lysates from total cell extracts were subjected to SDS-PAGE and immunoblotted with an anti-PAR specific antibody.


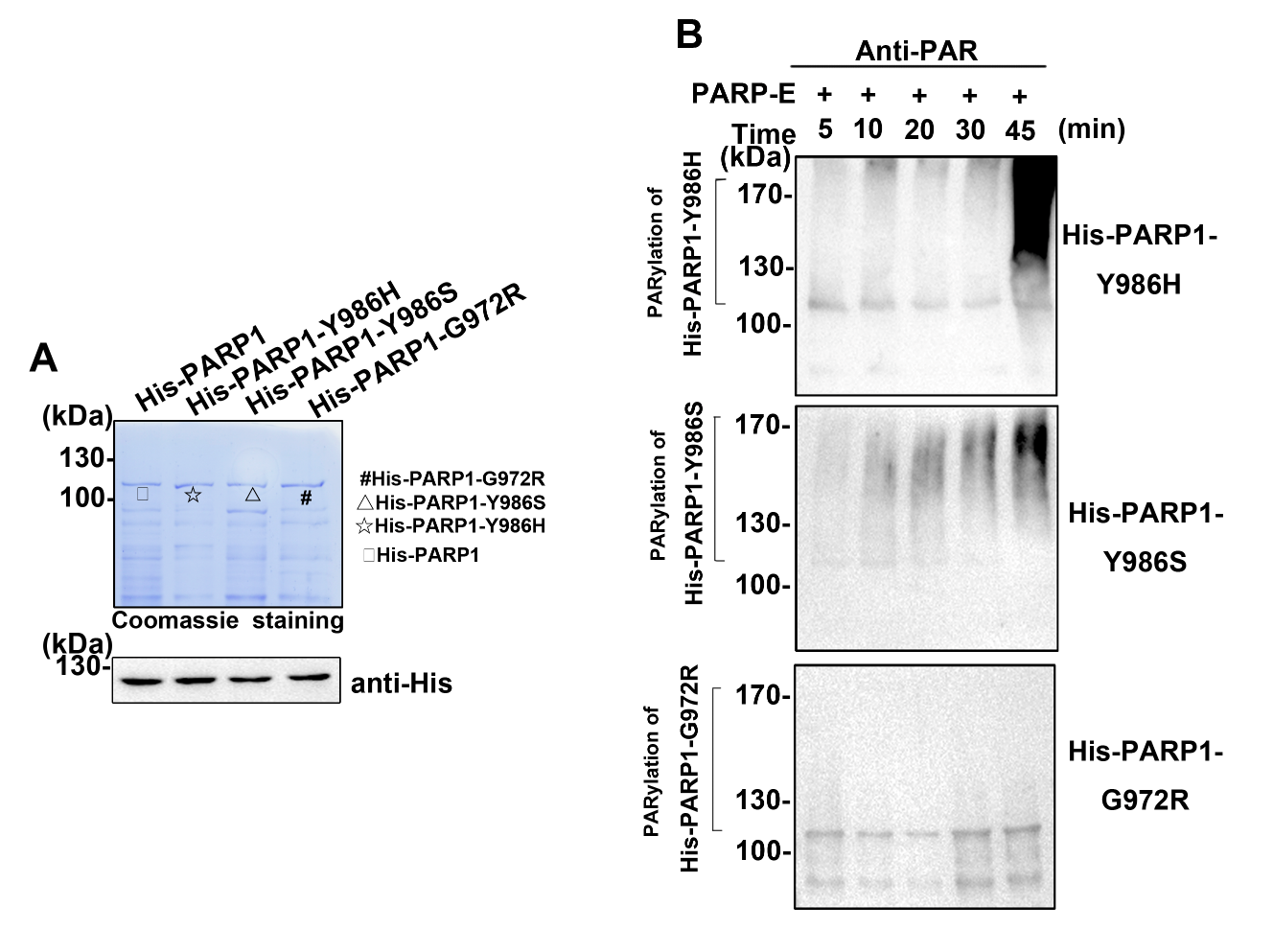


**Fig.S4 Biochemical characterization of different PARP1 variants**

**(A)** The respective PARP1 variants proteins are purified using an *E. coli* expression system. His-PARP1, His-PARP1-G972R, His-PARP1-Y986H and His-PARP1-Y986S were purified from bacteria (visualized by coomassie brilliant blue staining, upper panel) and immunoblotted with an anti-PARP1 specific antibody (lower panel).

**(B)** Time-dependent automodification reaction of different PARP1 variants. His-PARP1, His-PARP1-G972R, His-PARP1-Y986H and His-PARP1-Y986S were purified from bacteria, and then incubated with PARP-enzyme (PARP-E) for different 5, 10, 20, 30, 45 min and then subjected to immunoblotting to detect PARylation levels.
