# Supplementary material for: Poly (ADP-ribose) polymerase 1 promotes HuR/ELAVL1 cytoplasmic localization and inflammatory gene expression by regulating p38 MAPK activity
Source: Cell Mol Life Sci. 2024 Jun 9;81(1):253. doi: 10.1007/s00018-024-05292-2 (PMC11335290; doi:10.1007/s00018-024-05292-2)

1A

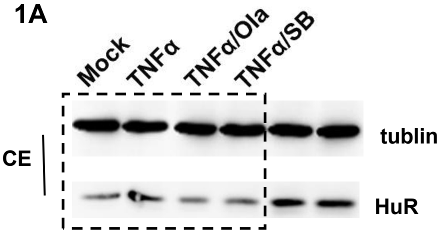

1B

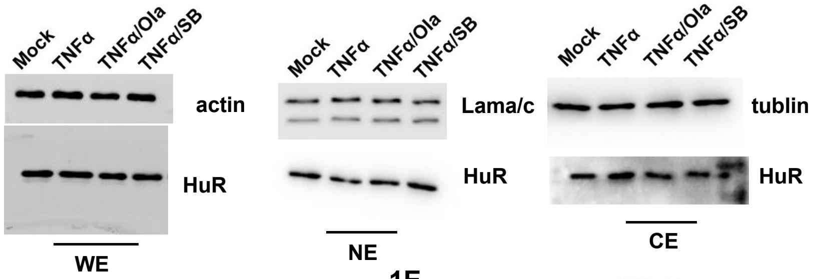

1C

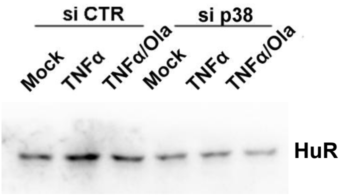

1D

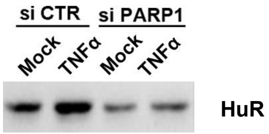

1E

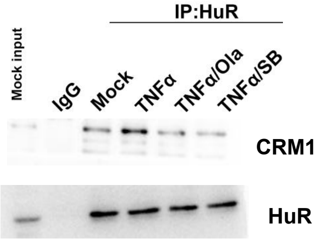

The band densitometry of p38 , normalized over siCTR-p38 from three independent experiments.

				P
si CTR	1	1	1	0
	1.142196	0.956359	1.049278	0.131407
	1.080284	0.941275	1.01078	0.098294
si p38	0.572704	0.387739	0.480222	0.13079
	0.409139	0.466796	0.437968	0.04077
	0.466222	0.569015	0.517618	0.072685

The band densitometry of PARP1 , normalized over siCTR-PARP1 from three independent experiments.

				P
si CTR	1	1	1	0
	1.700592	1.481617	1.591105	0.154839
si PARP1	0.604111	0.404299	0.504205	0.141288
	0.744704	0.526366	0.635535	0.154388

The band densitometry of CRM1 , normalized over Mock from three independent experiments.

				P
Mock	1	1	1	0
TNFα	2.139165	2.36751	2.253337	0.161465
TNFα/Ola	0.888555	0.834528	0.861541	0.038203
TNFα/SB	0.598657	0.516437	0.557547	0.058138

1F

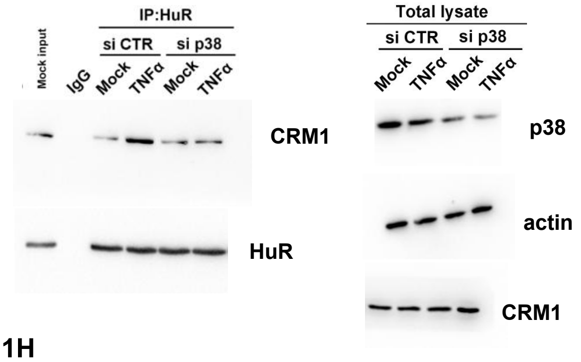

1G

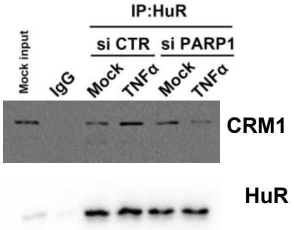

1H

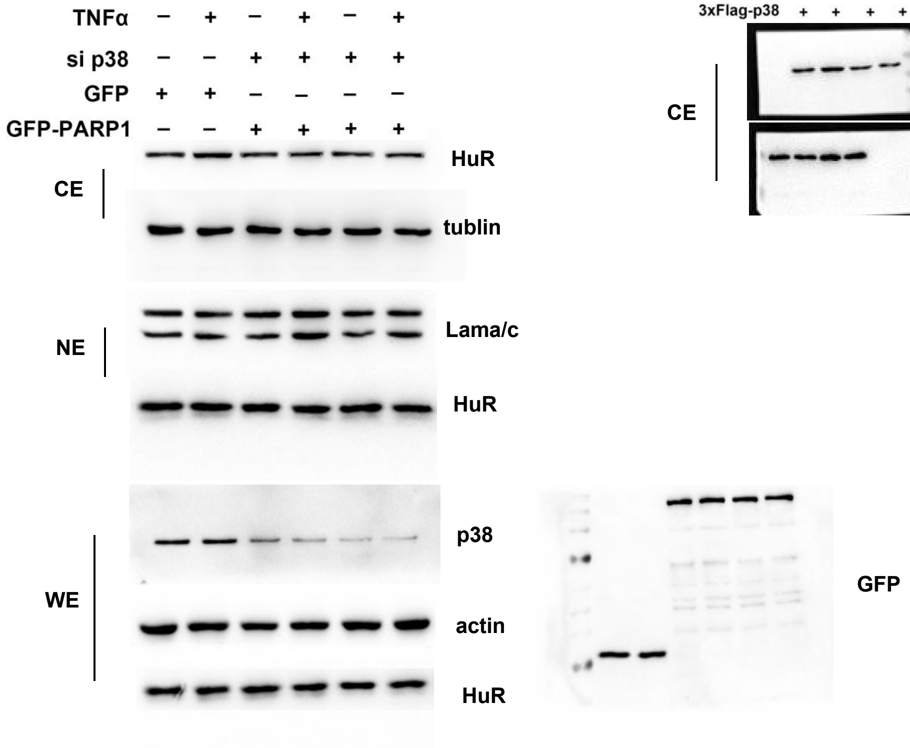

1I

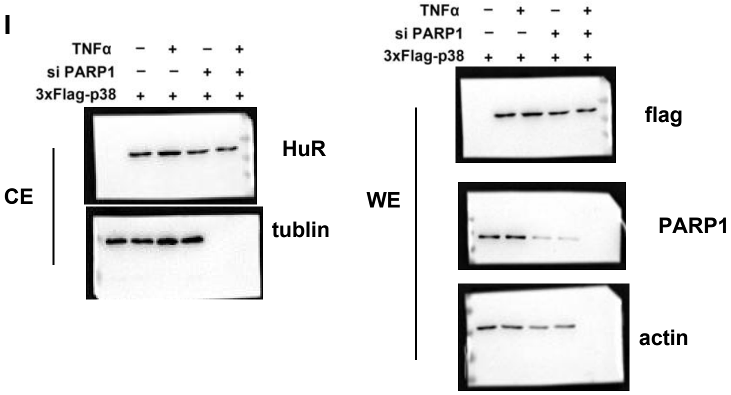

2A

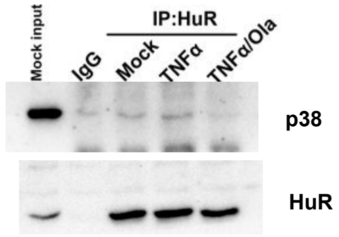

The band densitometry of p38 ,
normalized over Mock
from three independent experiments.

				P
Mock	1	1	1	0
TNFα	1.66108	1.41303	1.537055	0.175398
TNFα/Ola	0.83085	0.72635	0.7786	0.073893

2B

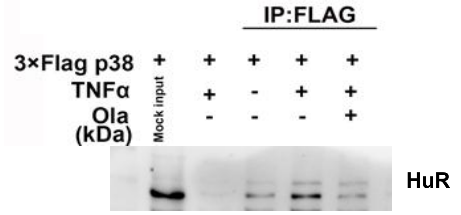

The band densitometry of HuR ,
normalized over Mock
from three independent experiments.

				P
Mock	1	1	1	0
TNFα	2.552819	2.037015	2.294917	0.364728
TNFα/Ola	0.711001	0.878385	0.794693	0.118358

2C

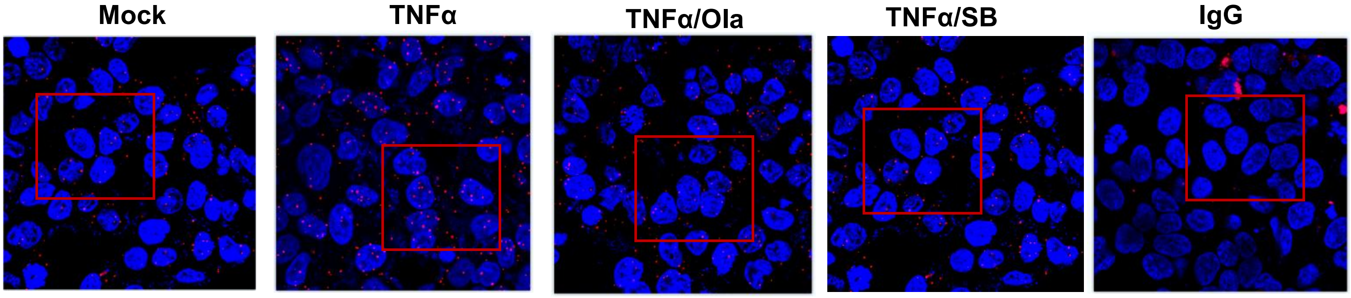

2D

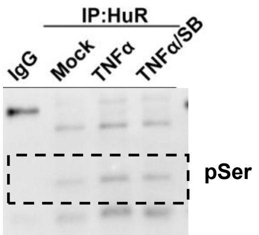

The band densitometry of p-Ser ,
normalized over Mock
from three independent experiments.

				P
Mock	1	1	1	0
TNFα	3.419352	2.762154	3.090753	0.464709
TNFα/SB	1.709716	1.564314	1.637015	0.102815

2E

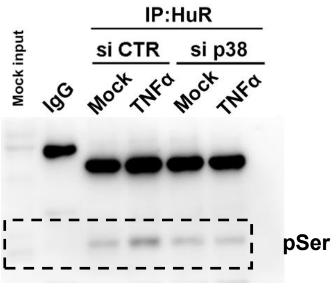

The band densitometry of p-Ser ,
normalized over Mock
from three independent experiments.

					P
si CTR	Mock	1	1	1	0
	TNFα	2.883979	2.788405	2.836192	0.06758
si p38	Mock	1.166142	1.320273	1.243208	0.108987
	TNFα	0.820206	1.016982	0.918594	0.139142

2H

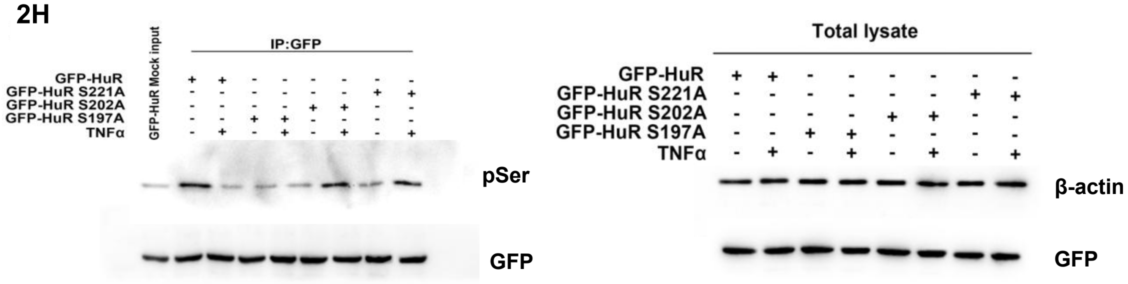

2I

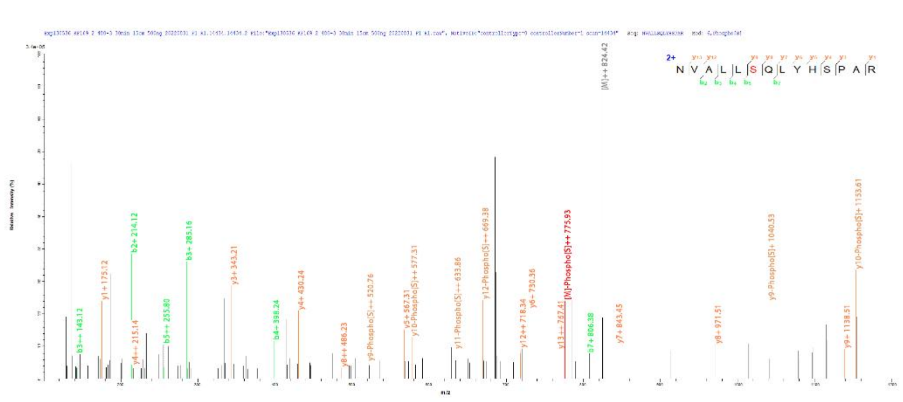

3A

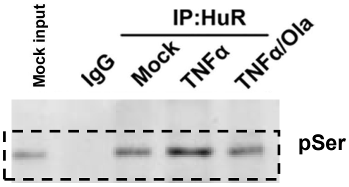

The band densitometry of p-Ser, normalized over Mock from three independent experiments.

				P
Mock	1	1	1	0
TNFα	3.1187167	2.6276461	2.8731814	0.3472394
TNFα/Ola	1.238108	1.0776629	1.1578854	0.1134518

3B

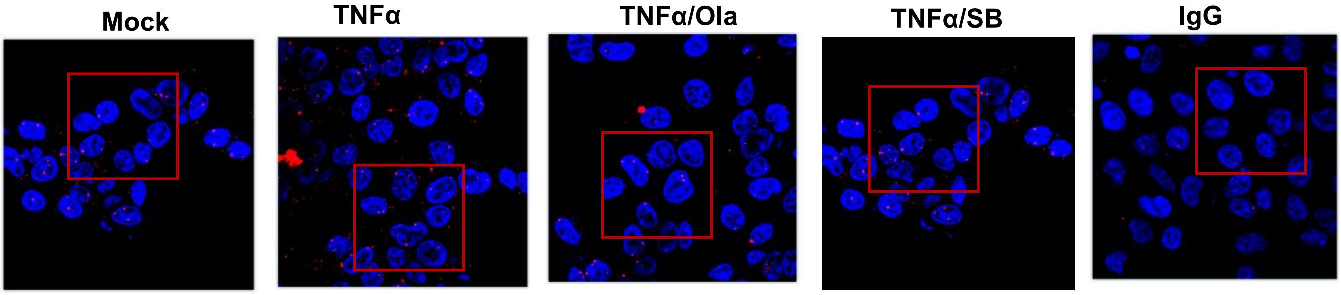

3C

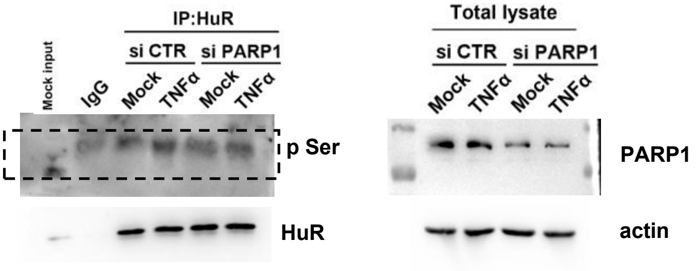

The band densitometry of p-Ser, normalized over Mock from three independent experiments.

						P
si CTR	Mock	1	1	1	0
	TNFα	1.887241	1.572578	1.72991	0.222501
si PARP1	Mock	0.878321	1.023625	0.950973	0.102746
	TNFα	0.979949	1.223831	1.10189	0.17245

3D

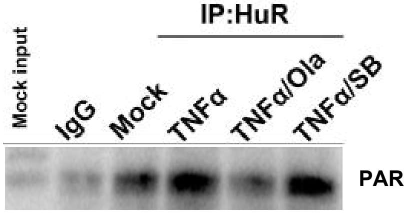

3E

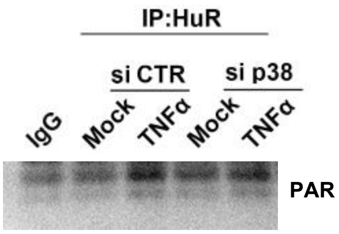

3F

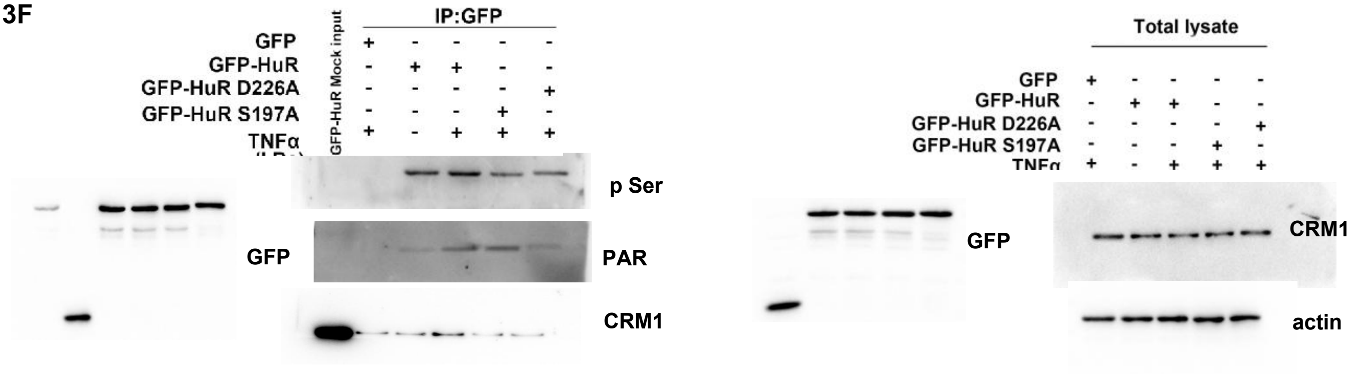

3G

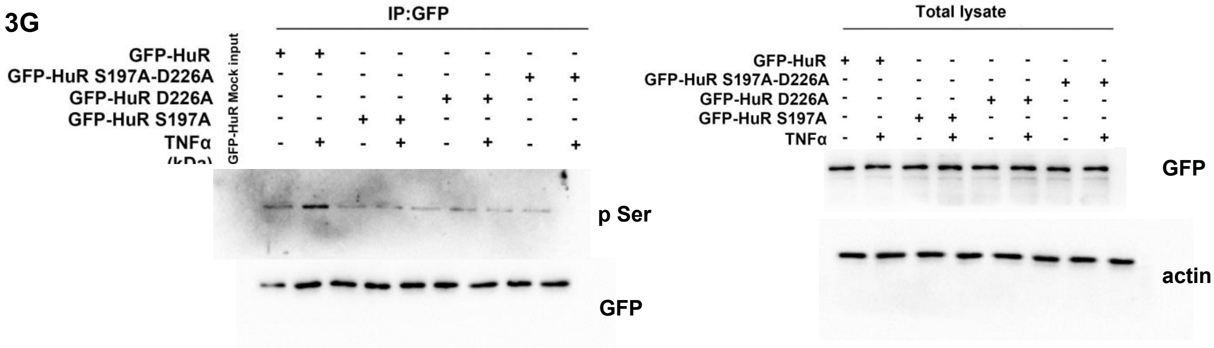

4A

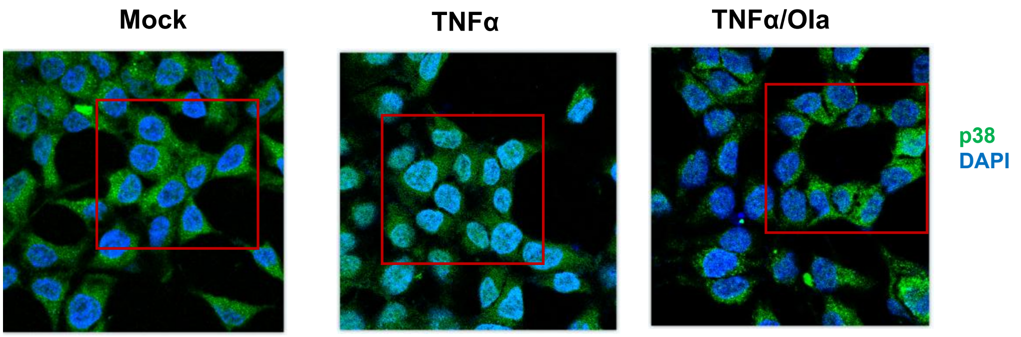

4B

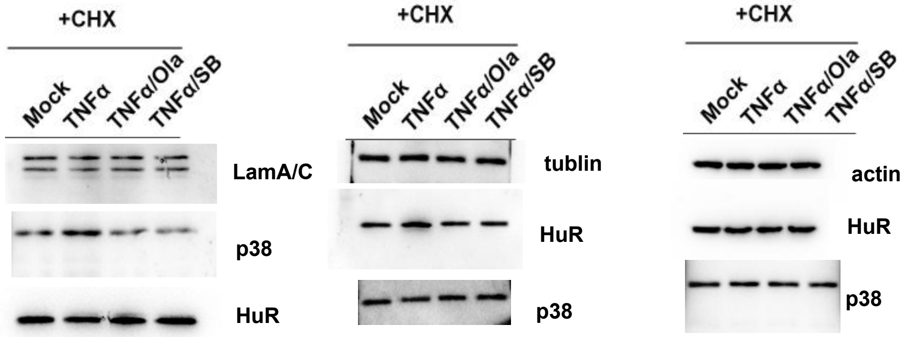

4D

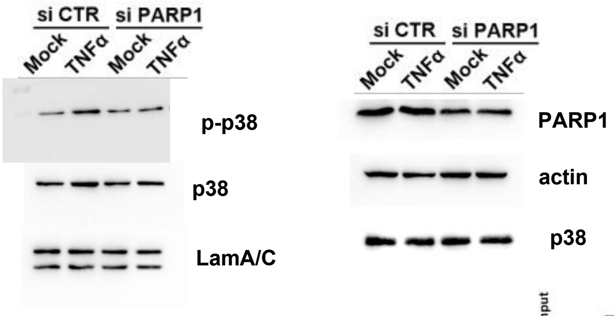

4E

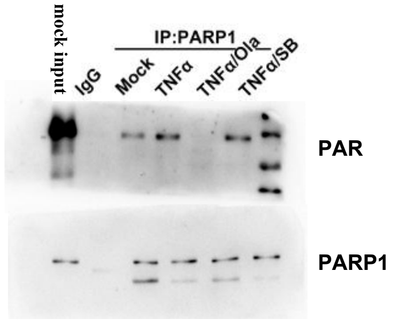

4F

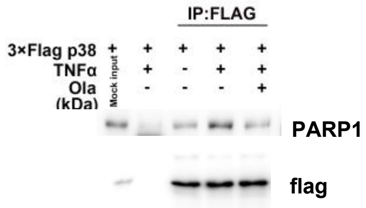

4G

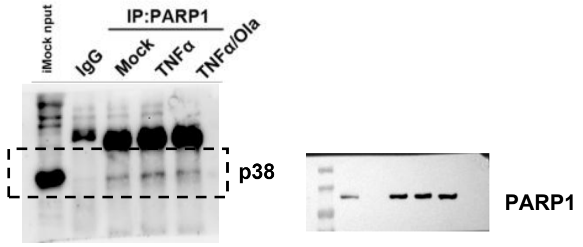

The band densitometry of PARP1 , normalized over Mock from three independent experiments.

				P
Mock	1	1	1	0
TNFα	1.551414	1.491809	1.521611	0.042147
TNFα/Ola	0.599868	0.711615	0.655742	0.079017

The band densitometry of p38 , normalized over Mock from three independent experiments.

				P
Mock	1	1	1	0
TNFα	1.468858	1.771176	1.620017	0.21377
TNFα/Ola	0.742949	0.975377	0.859163	0.164351

4H

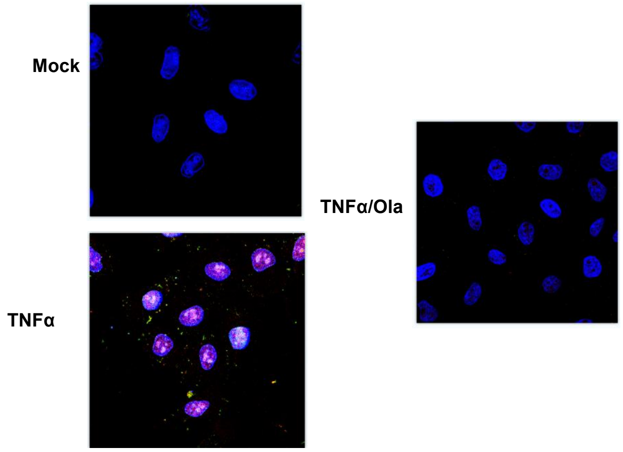

4K

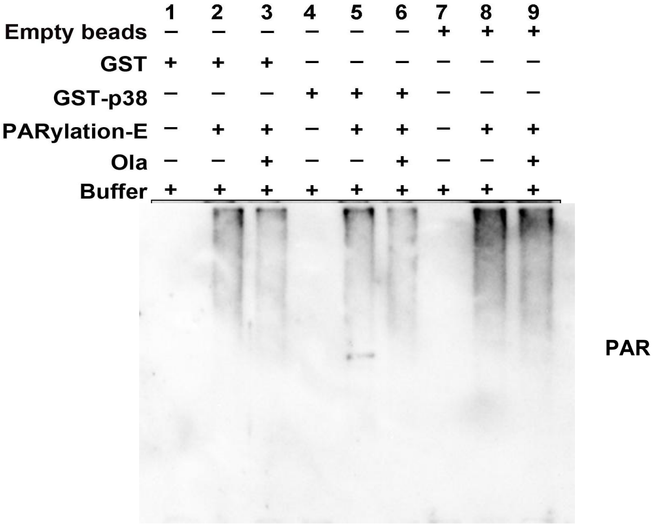

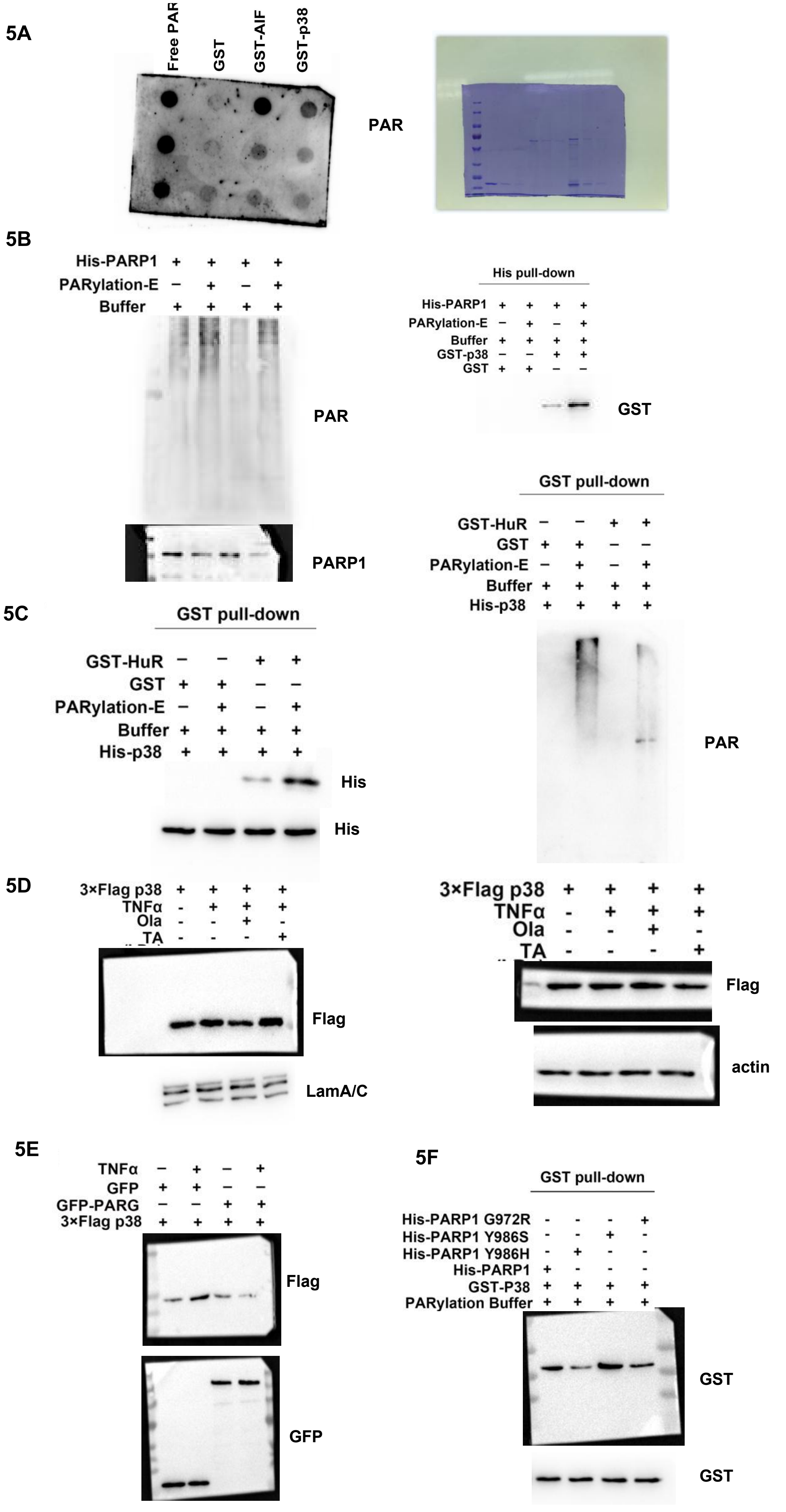
5A
Free PAR
GST
GST-AIF
GST-p38
PAR
5B
His-PARP1 + + + +
PARylation-E − + − +
Buffer + + + +
PAR
PARP1
His pull-down
His-PARP1 + + + +
PARylation-E − + − +
Buffer + + + +
GST-p38 − − + +
GST + + − −
GST
GST pull-down
GST-HuR − − + +
GST + + − −
PARylation-E − + − +
Buffer + + + +
His-p38 + + + +
PAR
5C
GST pull-down
GST-HuR − − + +
GST + + − −
PARylation-E − + − +
Buffer + + + +
His-p38 + + + +
His
His
5D
3×Flag p38 + + + +
TNFα - + + +
Ola - - + -
TA - - - +
Flag
LamA/C
3×Flag p38 + + + +
TNFα - + + +
Ola - - + -
TA - - - +
Flag
actin
5E
TNFα − + − +
GFP + + − −
GFP-PARG − − + +
3×Flag p38 + + + +
Flag
GFP
5F
GST pull-down
His-PARP1 G972R - - - +
His-PARP1 Y986S - - + -
His-PARP1 Y986H - + - -
His-PARP1 + - - -
GST-P38 + + + +
PARylation Buffer + + + +
GST
GST

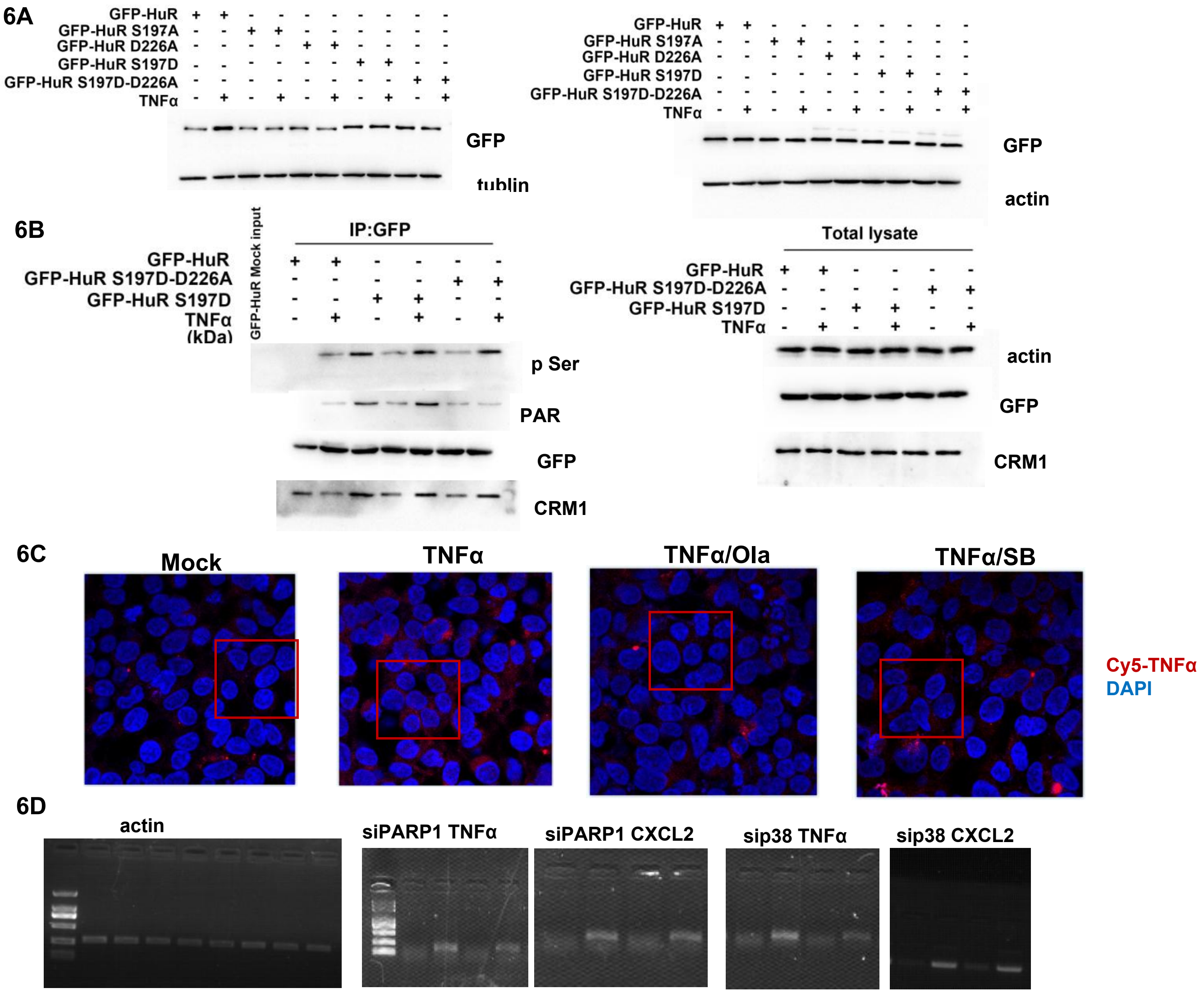

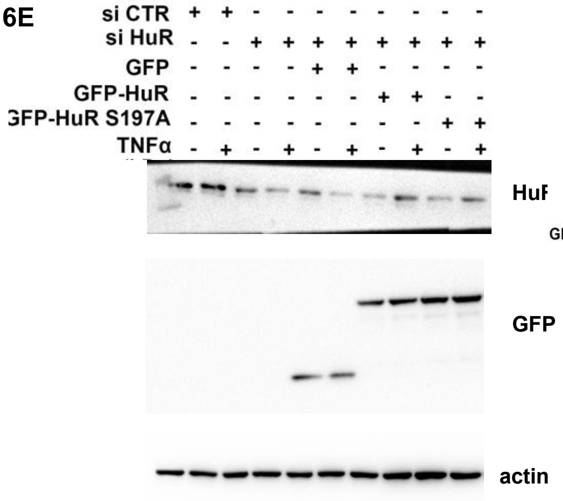

si CTR + + - - - - - - - -
si HuR - - + + + + + + + +
GFP - - - - + + - - - -
GFP-HuR - - - - - - + + - -
GFP-HuR S197A - - - - - - - - + +
TNFα - + - + - + - + - +

TNF

1	12.47496233	1	2.595531792	1	3.942540033	1	8.910813556	1	2.36744351
1	15.554	1	2.899	1	4.301	1	11.228	1	2.696
1	14	1	2.632	1	3.877	1	10.209	1	2.665

CXCL1

1	41.44395527	1	3.577141297	1	5.114411331	1	23.58349667	1	3.583339605
1	50.443	1	4.575	1	6.554	1	30.136	1	4.778
1	49.324	1	4	1	5.998	1	29.996	1	4.208

CXCL2

1	31.13378695	1	4.590134701	1	6.989183848	1	19.99433474	1	4.43600062
1	37.331	1	5.095	1	9.996	1	20.229	1	5.331
1	28.912	1	4.221	1	7.534	1	18.675	1	4.701

6F

CXCL2 t1/2

	GFP-HuR	GFP-HuR S197D	GFP-HuR D226A	GFP-HuR S197A	GFP-HuR S197D D226A
0h	1	1	1	1	1
2h	0.5732	0.68	0.2893	0.1788	0.69
4h	0.6032	0.66	0.26	0.14	0.71

TNFα t1/2

	GFP-HuR	GFP-HuR S197D	GFP-HuR D226A	GFP-HuR S197A	GFP-HuR S197D D226A
0h	1	1	1	1	1
2h	0.6065	0.7534	0.1698	0.216	0.72
4h	0.62	0.72	0.14	0.2	0.69

F

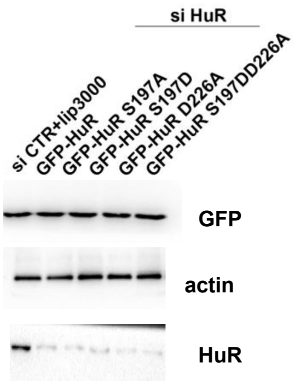

Supplement: Supplementary file 2 — Supplementary file2 (PDF 1351 KB) [file 18_2024_5292_MOESM2_ESM.pdf]
